# Targeting ASIC1a Promotes Neural Progenitor Cell Migration and Neurogenesis in Ischemic Stroke

**DOI:** 10.34133/research.0105

**Published:** 2023-06-01

**Authors:** Hongfei Ge, Tengyuan Zhou, Chao Zhang, Yupeng Cun, Weixiang Chen, Yang Yang, Qian Zhang, Huanhuan Li, Jun Zhong, Xuyang Zhang, Hua Feng, Rong Hu

**Affiliations:** ^1^ Department of Neurosurgery and Key Laboratory of Neurotrauma, Southwest Hospital, Third Military Medical University (Army Medical University), 400038 Chongqing, China.; ^2^ Medical Research Center, Southwest Hospital, Third Military Medical University (Army Medical University), 400038 Chongqing, China.; ^3^ Pediatric Research Institute, Ministry of Education Key Laboratory of Child Development and Disorders, National Clinical Research Center for Child Health and Disorders, Children’s Hospital of Chongqing Medical University, 400014 Chongqing, China.

## Abstract

Cell replacement therapy using neural progenitor cells (NPCs) has been shown to be an effective treatment for ischemic stroke. However, the therapeutic effect is unsatisfactory due to the imbalanced homeostasis of the local microenvironment after ischemia. Microenvironmental acidosis is a common imbalanced homeostasis in the penumbra and could activate acid-sensing ion channels 1a (ASIC1a), a subunit of proton-gated cation channels following ischemic stroke. However, the role of ASIC1a in NPCs post-ischemia remains elusive. Here, our results indicated that ASIC1a was expressed in NPCs with channel functionality, which could be activated by extracellular acidification. Further evidence revealed that ASIC1a activation inhibited NPC migration and neurogenesis through RhoA signaling-mediated reorganization of filopodia formation, which could be primarily reversed by pharmacological or genetic disruption of ASIC1a. In vivo data showed that the knockout of the ASIC1a gene facilitated NPC migration and neurogenesis in the penumbra to improve behavioral recovery after stroke. Subsequently, ASIC1a gain of function partially abrogated this effect. Moreover, the administration of ASIC1a antagonists (amiloride or Psalmotoxin 1) promoted functional recovery by enhancing NPC migration and neurogenesis. Together, these results demonstrate targeting ASIC1a is a novel strategy potentiating NPC migration toward penumbra to repair lesions following ischemic stroke and even for other neurological diseases with the presence of niche acidosis.

## Introduction

Ischemic stroke, accounting for about 85% of all strokes, usually causes death and life-long disability for suffers worldwide [1–3]. Although many therapeutic strategies have been developed to facilitate functional recovery, the majority of stroke patients have to seek medical assistance from professionals due to unsatisfactory functional recovery [4]. Neural progenitor cells (NPCs), with the ability of migrating from the subventricular zone (SVZ) toward infarct to promote neurogenesis, seem to be promising to facilitate functional recovery after ischemic stroke [5–8]. However, only a small number of SVZ-derived NPCs migrate toward the infarct and usually differentiate into astrocytes to form glial scar [2,7]. Due to the nonpermissive microenvironment following ischemic stroke, the insufficient number of NPCs migrating toward infarct restricts neurogenesis in penumbra. Therefore, deciphering factors inhibiting NPC migration and neurogenesis in penumbra might serve as a feasible therapeutic strategy after ischemic stroke.

Microenvironmental acidosis resulting from the accumulation of extracellular protons is a common imbalanced homeostasis following ischemic stroke [9,10], which not only exerts devastating disturbance to microenvironment but also causes damage to brain tissue around the infarct necrotic core (the ischemic penumbra). Acid-sensing ion channels (ASICs), which are proton-gated members belonging to the epithelial sodium channel/degenerin superfamily, could be activated by microenvironmental acidosis [11–13]. To date, researchers have found that 4 separate genes (ASIC1, ASIC2, ASIC3, and ASIC4) encode 7 ASIC subunits (ASIC1a, ASIC1b1, ASIC1b2, ASIC2a, ASIC2b, ASIC3, and ASIC4) in central and peripheral nervous systems of mammals [9,14,15]. ASIC1a is predominantly expressed in neurons of central nervous systems (CNSs) [14,16], mainly in the form of ASIC1a homomers and ASIC1a/2a [17] and ASIC1a/2b heteromers [18]. Meanwhile, previous studies have indicated that activating ASIC1a by sustained tissue acidosis markedly contributes to ischemic injury, whereas the blockage of ASIC1a reduces infarct volume after ischemic stroke and exerts neuroprotective effects following progressive multiple sclerosis [12,19]. Furthermore, our previous study shows that ASIC1a activation exaggerates secondary injury after traumatic spinal cord injury (SCI), and the down-regulation of ASIC1a substantially decreases tissue damage and facilitates functional restoration after SCI [20]. Given that NPCs are the primary regenerative cells after CNS injury, exploring the effect of ASIC1a on NPCs and unraveling the relevant mechanism might fill a critical gap and provide a therapeutic target after ischemic stroke.

Here, we showed that ASIC1a was expressed in NPCs with channel functionality. ASIC1a activation by acidic stimulation impaired migration and neurogenesis of NPCs. Subsequently, the bulk RNA sequencing (RNA-seq) was implemented to screen the downstream effectors of ASIC1a, and the relevant mechanism was investigated. We found that activating ASIC1a suppressed NPC migration via triggering the RhoA signaling pathway to attenuate filopodia formation. Further evidence demonstrated that ASIC1a knockdown or the application of ASIC1a antagonists facilitates NPC migration toward the peri-infarct and neurogenesis, therefore promoting functional recovery after ischemic stroke. These findings reveal that ASIC1a plays a vital role in regulating NPCs, which provides an intervention target for optimizing stem cell therapy using ASIC1a antagonists after ischemic stroke and even for other neurological diseases with the presence of niche acidosis.

## Results

### ASIC1a is expressed in NPCs with channel functionality

**Fig. 1.**
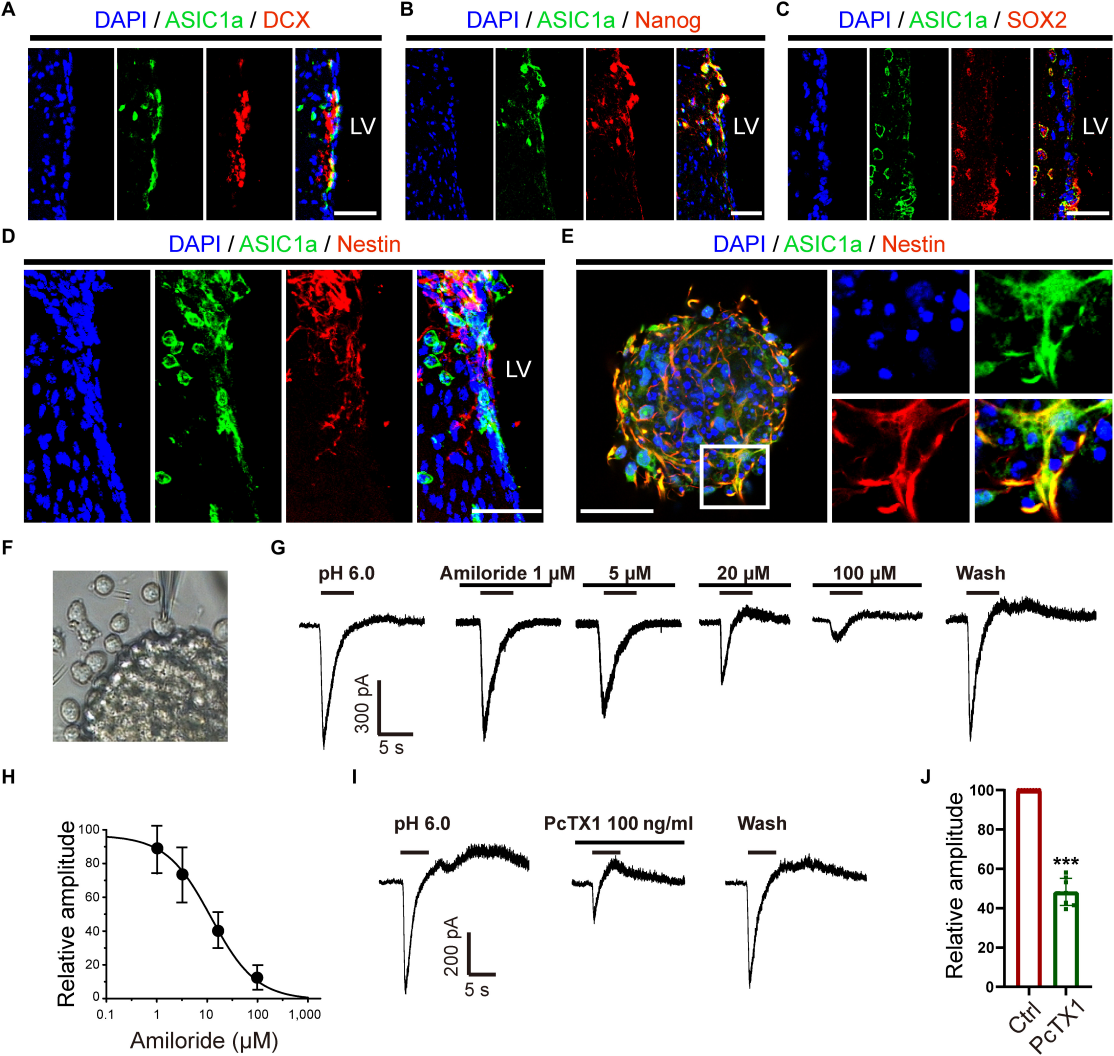
ASIC1a is expressed in NPCs. (A to D) Immunostaining images illustrating colocalization of ASIC1a and DCX (A), Nanog (B), SOX2 (C), and Nestin (D) in SVZ. Scale bars, 20 μm. (E) Immunostaining images depicting colabeling of ASIC1a and Nestin in the cultured neurospheres. The square insets demonstrate the filopodia formation at the edge of cytoskeleton in each group. Scale bar, 50 μm. (F and G) The representative picture (F) and traces (G) showing pH 6.0-induced ASIC currents and the inhibitory effect of amiloride on the ASIC currents on a migrating NPC out from the neurosphere using the whole-cell patch clamp. (H) The curve showed that amiloride dose-dependently inhibited the ASIC-like currents in NPCs with a half-maximal inhibitory concentration of 11.98 μM. (I and J) Typical traces of pH 6.0-induced currents (I) and the inhibitory effect of PcTX1 (J) on the ASIC currents. DAPI was used to counterstain the nuclei. ***P < 0.001.

To determine whether ASIC1a is expressed in NPCs around lateral ventricle (LV), the immunostaining of ASIC1a with different markers of NPCs was performed. The immunostaining images depicted that ASIC1a was colocalized with doublecortin (DCX) (Fig. 1A), Nanog (Fig. 1B), SOX2 (Fig. 1C) and Nestin (Fig. 1D), indicating that ASIC1a was expressed in NPCs around LV in adult mouse brain. Subsequently, the adult NPCs were cultured, and the neurospheres were grown in floating condition on day 3 in enrichment medium (Fig. S1A), and most of the cultured cells expressed Nestin (Fig. S1B). Then, the multipotency of NPCs was determined by immunostaining, and the images indicated that the cultured NPCs held the potential of differentiating into glial fibrillary acidic protein-positive (GFAP^+^) (Fig. S1C), DCX^+^ (Fig. S1D), and Olig2^+^ cells (Fig. S2E). Moreover, the immunostaining images revealed that ASIC1a was colabeled with Nestin^+^ cells isolated from SVZ-derived neurospheres (Fig. 1E). Collectively, these findings suggest that ASIC1a is expressed in NPCs.

Afterwards, ASIC currents were functionally characterized in NPCs by whole-cell patch clamp. Large transient inward currents were triggered by extracellular acidosis of pH 6.0, which could be blocked by amiloride, a common nonspecific inhibitor for ASICs [18], in a dose-dependent manner with a half-maximal inhibitory concentration of 11.98 ± 1.04 μM (Fig. 1F to H). Furthermore, 100 ng/ml Psalmotoxin 1 (PcTX1), which specifically inhibits homomeric ASIC1a and ASIC1a/2b channels [18], could decrease the ASIC1a amplitude to 48.26 ± 5.25% (Fig. 1I and J). The inhibitory effects of the 2 inhibitors were almost abrogated after washout (Fig. 1G and I), providing further evidence that these currents were ASIC currents. The above results suggest that ASIC1a is expressed in NPCs and is capable of forming functional channels, which is sensitive to acidic stimulation.

### ASIC1a activation induced by acidification inhibits NPC migration and neurogenesis in vitro

The above findings indicated NPCs expressed ASIC1a and ASIC1a could be specifically activated by extracellular acidification [14,21]. Then, the effect of ASIC1a activation on the survival of NPCs using Annexin-V/propidium iodide (PI) staining, lactate dehydrogenase (LDH) releasing and Cell Counting Kit-8 (CCK8) assays under different pH values (pH 7.4, pH 7.0, pH 6.5, and pH 6.0). The percentage of apoptotic NPCs using Annexin-V/PI staining and the level of LDH releasing showed no differences among pH 7.4, pH 7.0, and pH 6.5, which were obviously increased under pH 6.0 at 6, 12, and 24 h (Fig. S2A to C). In addition, CCK8 assays indicated that cell viability was apparently reduced under pH 6.0 at 24 h, whereas no distinction was detected among pH 7.4, pH 7.0, and pH 6.5 (Fig. S2D). Hence, ASIC1a activation using extracellular acidification exerted no evident influence on the survival and viability of NPCs, but severe acidosis resulted in decreasing survival and viability of NPCs. Therefore, the acidic condition in the following in vitro experiments was selected as pH 6.5, which could not affect survival and viability but could activate ASIC1a in NPCs.

**Fig. 2.**
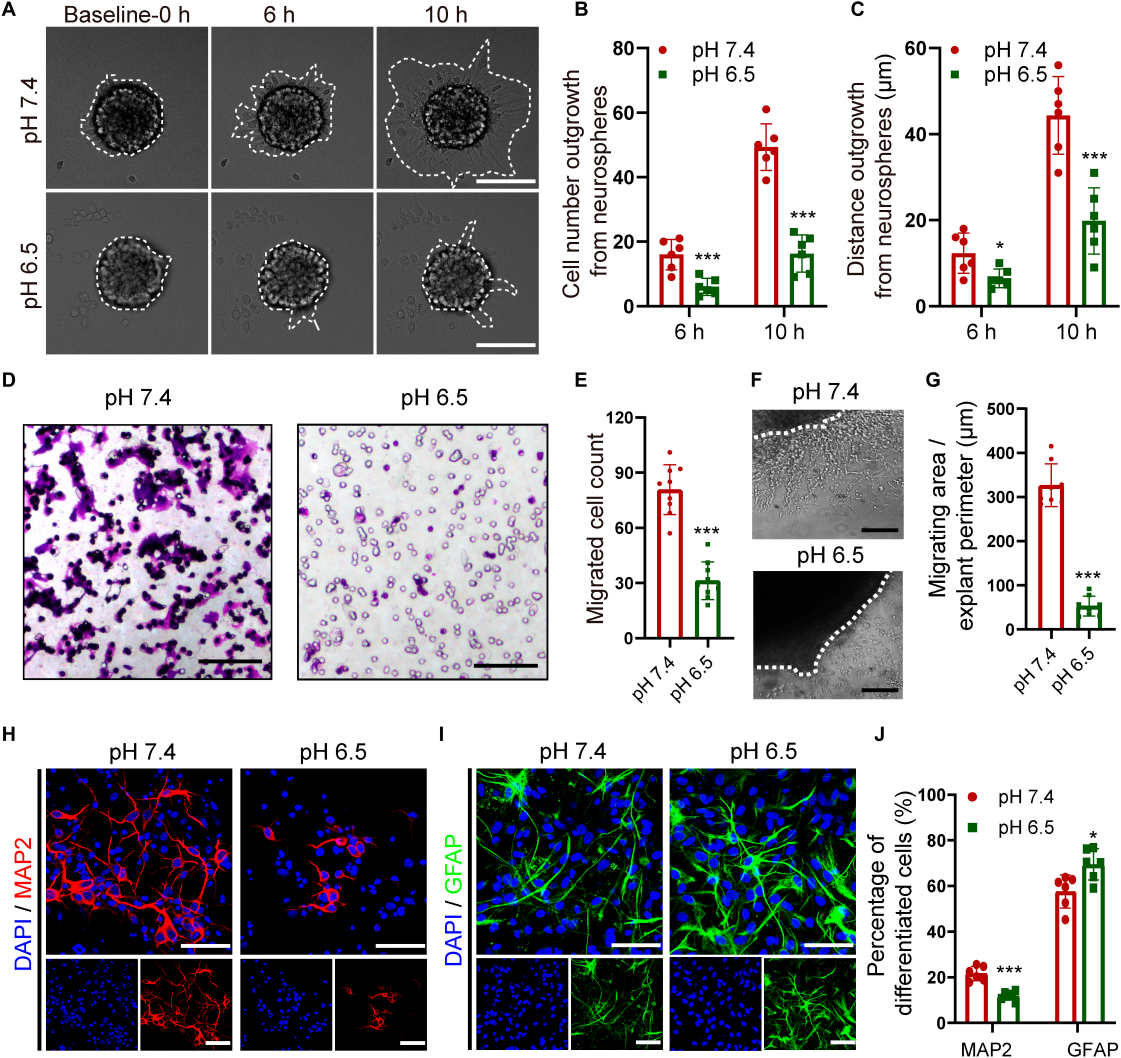
ASIC1a activation induced by acidosis inhibits NPC migration and neurogenesis. (A) The migration of cultured neurospheres under different pH conditions at different time points. Scale bars, 100 μm. (B and C) Quantification of the migrating cell number (B) and distance (C) from (A). (D and E) Representative pictures of transwell assays (D) and quantitation (E) showing the migration of NPCs under different pH conditions. Scale bars, 20 μm. (F and G) Representative images (F) and quantification (G) of cell emigration from the SVZ explants under different pH conditions. Scale bars, 100 μm. (H and I) Representative immunostaining images of MAP2 (H) and GFAP (I) showing the differentiation of NPCs under different pH conditions. Scale bars, 10 μm. (J) Bar chart showcasing the percentage of MAP2^+^ and GFAP^+^ cells from (H) and (I). DAPI was used to counterstain the nuclei. *P < 0.05, ***P < 0.001.

To clarify the role of ASIC1a in NPC migration, neurospheres were seeded on poly-L-ornithine (PLO)-precoated culture clusters under pH 7.4 and pH 6.5. The time-lapse phase-contrast images delineated that the number of migrating cells and distance out from neurospheres were significantly reduced under the acidic condition of pH 6.5 (Fig. 2A to C). Furthermore, Transwell assays showed that the number of cells migrating from upper to lower chambers was substantially decreased when the pH was reduced from pH 7.4 to pH 6.5 (Fig. 2D and E). In addition, SVZ explants were exposed to different pH culture media, and cells emigrated from the explants were obviously repressed in the pH 6.5 culture medium, compared to pH 7.4 (Fig. 2F and G). Finally, the effect of acidic condition on NPC differentiation was investigated using immunostaining. The results showcased that the percentage of MAP2^+^ cells derived from NPCs was obviously lowered at pH 6.5 compared to pH 7.4 (Fig. 2H to J). By contrast, the proportion of GFAP^+^ cells at pH 6.5 was profoundly higher than that at pH 7.4 (Fig. 2H to J). In summary, these data indicate that ASIC1a activation by extracellular acidosis impairs NPC migration and differentiation into neurons, thereby suppressing neurogenesis in vitro.

### ASIC1a plays a significant role in mediating NPC migration and differentiation into neurons under the acidic condition of pH 6.5

**Fig. 3.**
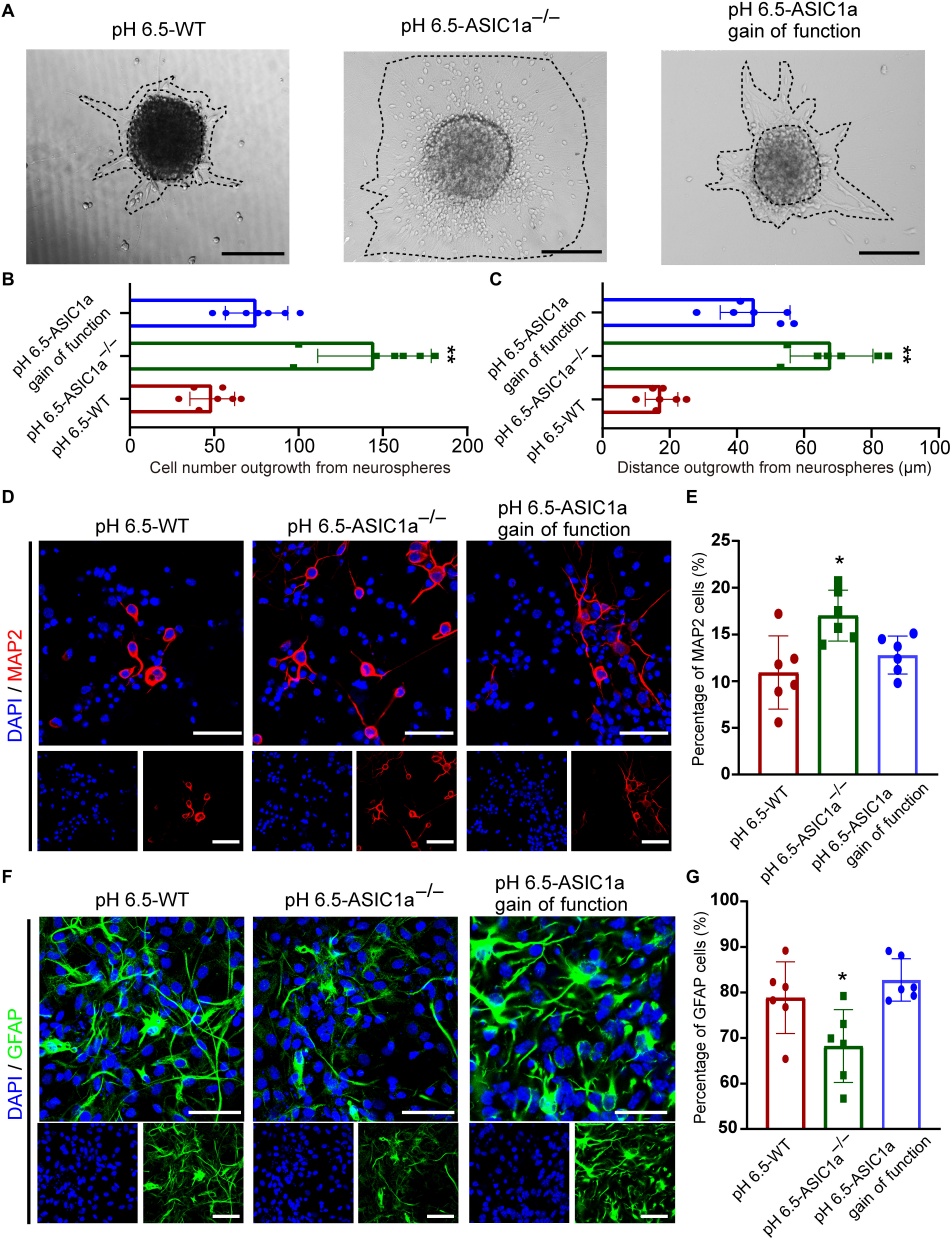
ASIC1a deletion promotes NPC migration and differentiation into neurons under the acidic condition of pH 6.5. (A to C) The migration of cultured neurospheres in different groups (WT, ASIC1a*^−/−^*, and ASIC1a gain of function) (A) and the migrating cell number (B) and distance (C) under pH 6.5 condition at 24 h. Scale bars, 100 μm. (D) Representative immunostaining images of MAP2 showing the differentiation of NPCs in different groups (WT, ASIC1a*^−/−^*, and ASIC1a gain of function) under pH 6.5 condition. Scale bars, 10 μm. (E) Bar graph indicating the percentage of MAP2^+^ cells from (D). (F) Typical immunostaining images of GFAP in different groups (WT, ASIC1a*^−/−^*, and ASIC1a gain of function) showing the differentiation of NPCs under pH 6.5 condition. Scale bars, 10 μm. (G) Bar graph indicating the percentage of GFAP^+^ cells from (F). DAPI was applied to counterstain the nuclei. *P < 0.05, **P < 0.01.

NPCs collected from ASIC1a*^−/−^* mice (Fig. S3) and ASIC1a gain-of-function NPCs using ASIC1a*^−/−^* NPCs transfected with ASIC1a-specific CRISPR (Fig. S4) were used to explore the role of ASIC1a in regulating migration and differentiation of NPCs under pH 6.5 condition. The number of migrating cells and distance out from neurospheres isolated from ASIC1a*^−/−^* mice were obviously increased under the acidic condition of pH 6.5, compared to the wild-type (WT) (Fig. 3A to C). However, ASIC1a gain of function partially reversed the effect of ASIC1a knockout on promoting NPC migration at pH 6.5 (Fig. 3A to C). Interestingly, the number of migrating cells and distance out from neurospheres isolated from ASIC1a*^−/−^* mice and WT mice at pH 7.4 showed no obvious difference (Fig. S5A and B). Moreover, the migrating area of SVZ explants from ASIC1a*^−/−^* mice was significantly increased than that from ASIC1a*^+/+^* mice at pH 6.5 (Fig. S6A and B). Subsequently, we examined the impact of ASIC1a knockout on cell differentiation. The percentage of MAP2^+^ cells derived from ASIC1a*^−/−^* NPCs was markedly higher than that from ASIC1a^+/+^ NPCs, while the proportion of GFAP^+^ cells derived from ASIC1a*^−/−^* NPCs was markedly lowered compared with that from ASIC1a^+/+^ NPCs (Fig. 3D to G). By contrast, ASIC1a gain of function counteracted the effect of ASIC1a knockout on the differentiation of NPCs (Fig. 3D to G).

### ASIC1a activation attenuates filopodia formation via triggering RhoA signaling pathway

**Fig. 4.**
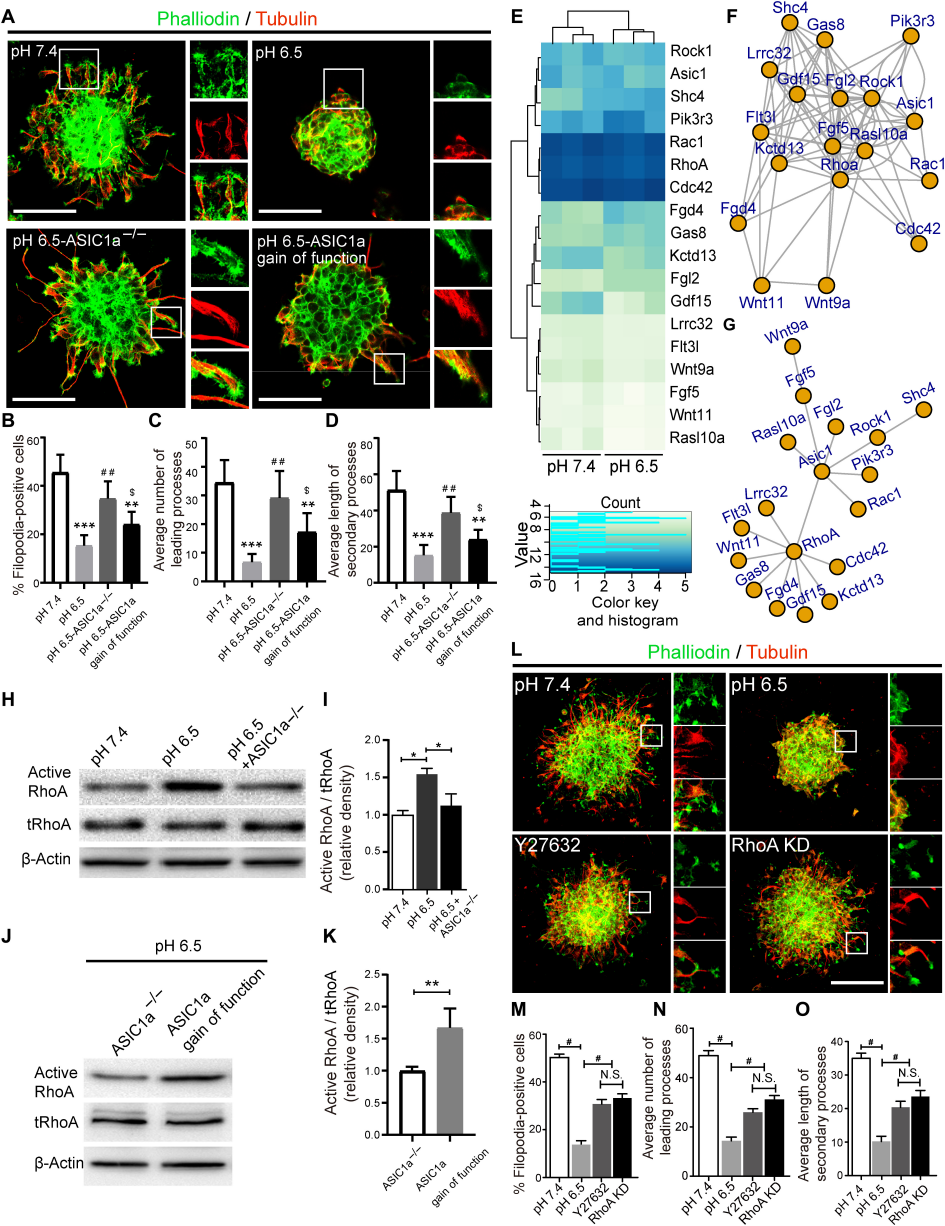
ASIC1a activation attenuate filopodia formation via triggering RhoA signaling pathway. (A to D) Typical images of filopodia and leading process formation in different groups (pH 7.4, pH 6.5, and pH 6.5 + ASIC1a*^−/−^*, pH 6.5 + ASIC1a gain of function) (A), and quantification of the average percentage of filopodia (B), number (C), and length (D) of leading processes from (A). The square insets showcased the filopodia formation at the edge of cytoskeleton in each group. DAPI was used to counterstain the nuclei. Scale bars, 50 μm. **P < 0.01, ***P < 0.001, compared with pH 7.4. ^##^*P* < 0.01, compared with pH 6.5. ^$^*P* < 0.05, compared with pH 6.5-ASIC1a*^−/−^*. (E) Heatmap of 18 genes from 165 DEG analysis in RNA-seq profile. (F) The coexpression network of the 18 genes from (E). (G) Minimum span tree of the coexpression network in (E). (H to K) Immunoblot bands demonstrating the expression (H and J) and semiquantitation (I and K) of active RhoA under different conditions. β-actin was used as an internal control. *P < 0.05, **P < 0.01. (L to O) Typical images of filopodia and leading processes formation in different groups (pH 7.4, pH 6.5, pH 6.5 + Y27632, and pH 6.5 + RhoA knockdown) (L), and quantification of the average percentage of filopodia (M), number (N), and length (O) of leading processes from (L). The square insets indicated the filopodia formation at the edge of cytoskeleton in each group. DAPI was used to counterstain the nuclei. Scale bars, 50 μm. ^#^*P* < 0.05. N.S., not significant.

Given that the cell migration needs membrane protrusion and is presumably determined by cytoskeletal reorganization [2,22,23], we investigated whether ASIC1a activation would influence the organization of cytoskeleton in NPCs. Therefore, phalloidin staining was employed to assess filopodia formation, a cell polarization indicator at the start of migration [23]. At the same time, the expression of tubulin, a symbol of cage-like microtubule structure [23], was also determined to assess the morphological structure of NPCs at 24 h under pH 6.5. We found that the percentage of filopodia formation was significantly decreased under the acidic condition of pH 6.5 compared with pH 7.4 (Fig. 4A and B), while the percentage of filopodia formation was significantly raised in ASIC1a*^−/−^* NPCs than in ASIC1a^+/+^ NPCs at pH 6.5 (Fig. 4A and B). Furthermore, the average number and length of leading processes were greater in ASIC1a*^−/−^* NPCs than in ASIC1a^+/+^ NPCs at pH 6.5 (Fig. 4A, C, and D). At the same time, ASIC1a gain of function reversed the effect of ASIC1a knockout on the percentage of filopodia formation, as well as average number and length of leading processes of NPCs under the acidic condition of pH 6.5 (Fig. 4A to D). Collectively, these results support the idea that ASIC1a activated by extracellular acidosis hinders NPC migration and neurogenesis by inhibiting filopodia formation, and ASIC1a deletion partially abrogated this effect by facilitating filopodia formation.

To elucidate the molecular mechanisms underlying ASIC1a activation-induced impairment of migration of NPCs, we performed RNA-seq to screen the pivotal downstream effectors when ASIC1a was activated by extracellular acidification. The de-expression of ASIC1 gene and 162 differential expressed genes (DEGs) were identified through RNA-seq analysis. Then, the related genes were selected based on pH and migration-related pathways to identify 18 interesting genes (Fig. 4E). The coexpression network analysis showed that ASIC1, RhoA, and Rac1 were 3 hub genes influencing NPC migration and differentiation (Fig. 4F). Next, the minimum span tree analysis demonstrated that RhoA was an evident effector of ASIC1 (Fig. 4G). Of note, the expression of active RhoA was remarkably elevated by Western blot assays under pH 6.5 condition compared to pH 7.4, whereas ASIC1a knockout reversed this effect (Fig. 4H and I). Simultaneously, we also found the expression of active RhoA was obviously decreased in neurospheres from ASIC1a*^−/−^* mice compared to ASIC1a gain-of-function mice at pH 6.5 (Fig. 4J and K). Together, these results demonstrate that RhoA is a crucial downstream effector of ASIC1a.

Afterward, a specific inhibitor of RhoA-dependent kinase Y27632 was administrated to provide further evidence that RhoA signaling was associated with the migration and differentiation of NPCs when ASIC1a was activated by acidification. The results demonstrated that the cell number of migrating cells and distance out from neurospheres were evidently increased with the administration of Y27632 under the acidic condition of pH 6.5 (Fig. S7A to C). Subsequently, a small interfering RNA (siRNA) targeting RhoA was incorporated into ASIC1a^+/+^ NPCs to further investigate the role of RhoA signaling in mediating NPC migration after ASIC1a was activated. The immunoblot bands depicted that the expression of RhoA was obviously decreased after NPCs were treated with RhoA siRNA (Fig. S7D). Then, the cell number of migrating cells and distance out from neurospheres were evidently increased with the application of RhoA siRNA at pH 6.5 (Fig. S7E to G). Simultaneously, the cytoskeletal organization and filopodia formation was partially counteracted by Y27632 or RhoA siRNA (Fig. 4L to O). Additionally, differentiation assay showed that Y27632 or RhoA siRNA increased the proportion of MAP2^+^ cells but decreased the percentage of GFAP^+^ cells derived from NPCs (data not shown). Collectively, these data indicate that ASIC1a activation triggers the RhoA signaling pathway to negatively regulate the migration and neurogenesis of NPCs under acidic conditions.

### ASIC1a deletion promotes functional recovery by facilitating NPC migration and neurogenesis in penumbra after ischemic stroke in mice

To verify whether ASIC1a activation affects the endogenous NPC migration, the model of distal middle cerebral artery occlusion (dMCAO), which has been proved to be a highly reproducible and well-defined infarct model without the confounding effects of reperfusion [24] and widely used in neurogenesis research [25], was established to mimic human stroke. In this study, male mice (~25 g, with congenic C57/BL6 background) were subjected to dMCAO.

**Fig. 5.**
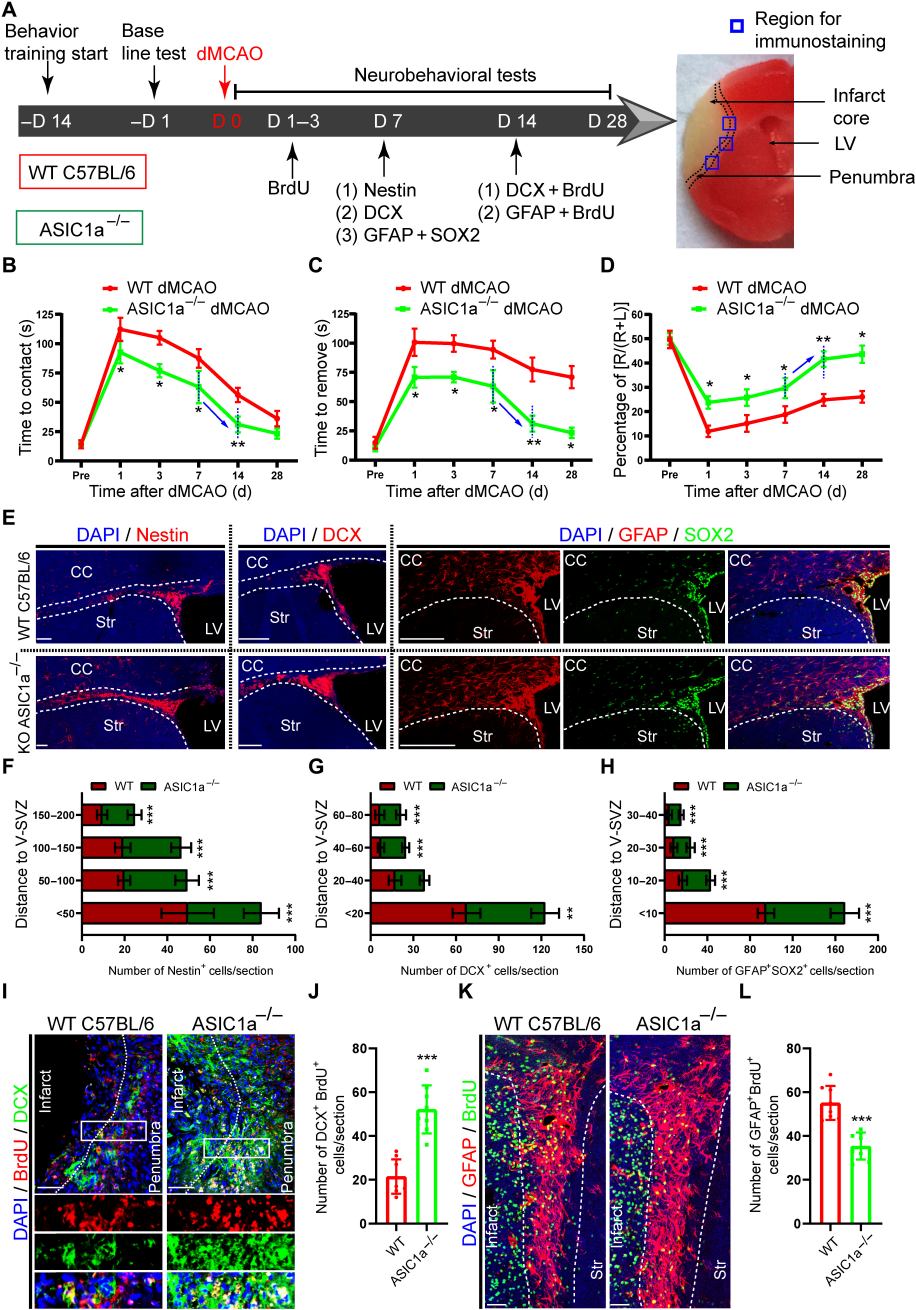
ASIC1a*^−/−^* mice exhibit better behavioral recovery through promoting neurogenesis in penumbra after ischemic stroke. (A) Experiment design and timeline. Behavior training was started 14 d before dMCAO. Baseline information was collected 1 d before dMCAO. Behavioral tests were conducted on days 1, 3, 7, 14, and 28. Immunostaining was performed to assess the migration of NPCs from SVZ to penumbra on day 7. Then, BrdU was administrated from day 1 to 3 post-dMCAO and immunostaining was implemented to evaluate the differentiation of migrated NPCs from V-SVZ to penumbra on day 14. The blue rectangle illustrated the region for immunostaining. (B to D) Summarized curves demonstrating the adhesive removal test (B and C) and corner test (D) on days 1, 3, 7, 14, and 28. (E) Representative immunostaining images of Nestin, DCX, and GFAP and SOX2 depicting the migration of NPCs from V-SVZ to penumbra on day 7. Dotted lines presented the margin of different brain regions. Scale bars, 20 μm. (F to H) Quantitation of Nestin^+^ cells (F), DCX^+^ cells (G), and GFAP^+^SOX2^+^ per section from (E) in each group. (I and J) Representative immunostaining images of DCX and BrdU (I), and quantitation (J) showing the differentiation of migrated NPCs into newborn neurons around infarct. The insets showcased the high magnification of DCX^+^BrdU^+^ cells from the rectangle at the infarct and penumbra in each group. Scale bars, 20 μm. (K and L) Typical immunostaining images of GFAP and BrdU (K), and quantification (L) demonstrating the differentiation of migrated NPCs into newborn astrocytes around infarct. DAPI was used to counterstain the nuclei. Scale bars, 20 μm. *P < 0.05, **P < 0.01, ***P < 0.001. LV, lateral ventricle; CC, callous corpus; Str, striatum.

Both WT and ASIC1a*^−/−^* mice were subject to dMCAO, and various assessments were performed to evaluate the role of ASIC1a in facilitating functional recovery (Fig. 5A). Firstly, the results demonstrated that ASIC1a*^−/−^* mice presented better behavioral recovery than WT mice using behavioral tests including adhesive removal test (Fig. 5B and C) and corner test (Fig. 5D) on days 1, 3, 7, 14, and 28 post-dMCAO. Intriguingly, a much sharper rise in behavioral improvement was observed from day 7 to 14 than other time points (blue arrows in Fig. 5B to D), implying that this period was a pivotal time window for neurorehabilitation in ASIC1a*^−/−^* mice. After that, the immunostaining of several markers of NPCs was implemented to assess the effect of ASIC1a ablation on NPC migration after dMCAO on day 7. The results showed that the number of Nestin^+^ cells in ASIC1a*^−/−^* mice was evidently increased along the route to penumbra at the distance from 50 to 200 μm, while that was obviously reduced less than 50 μm away from LV (Fig. 5E and F). Consistent with Nestin^+^ cells, the amount of DCX^+^ cells in ASIC1a*^−/−^* mice was prominently increased along the avenue to penumbra from 40 to 80 μm, whereas the amounts were markedly reduced less than 20 μm away from LV (Fig. 5E and G). Furthermore, the quantity of GFAP^+^ SOX2^+^ cells in ASIC1a*^−/−^* mice was profoundly increased along the path to penumbra from 10 to 40 μm, but that was significantly reduced less than 10 μm away from LV (Fig. 5E and H). These results demonstrate that ASIC1a deletion promotes NPC migration from SVZ to penumbra and functional restoration following ischemic stroke in mice.

To explore the effect of ASIC1a knockout on neurogenesis after ischemic stroke, we performed double immunostaining of DCX and 5-bromo-2-deoxyuridine (BrdU) among LV and along the rostral migratory stream (RMS). Unexpectedly, no significant difference was found in the number of BrdU^+^DCX^+^ cells around LV and along RMS between ASIC1a*^−/−^* and WT mice under normal physiological conditions (Fig. S8A to C), demonstrating that ASIC1a deletion did not affect the neurogenesis of NPCs in mice without dMCAO. Then, the BrdU pulse (continuous BrdU injections daily for 3 d post-dMCAO) was performed to evaluate the NPC differentiation in ischemic penumbra on day 14. The amount of BrdU^+^DCX^+^ cells in ASIC1a*^−/−^* mice was evidently increased in penumbra than that in WT mice (Fig. 5I and J). However, the quantity of BrdU^+^GFAP^+^ cells in ASIC1a*^−/−^* mice was significantly decreased in penumbra than that in WT mice (Fig. 5K and L). These data confirm that ASIC1a ablation facilitates NPC migration and neurogenesis in the penumbra after ischemic stroke in mice. These results suggest that knockout of the ASIC1a gene facilitated NPC migration and neurogenesis in the penumbra to improve behavioral recovery after stroke.

### ASIC1a gain of function partially abrogates the effect of ASIC1a deletion on potentiating NPC migration and neurogenesis after ischemic stroke

**Fig. 6.**
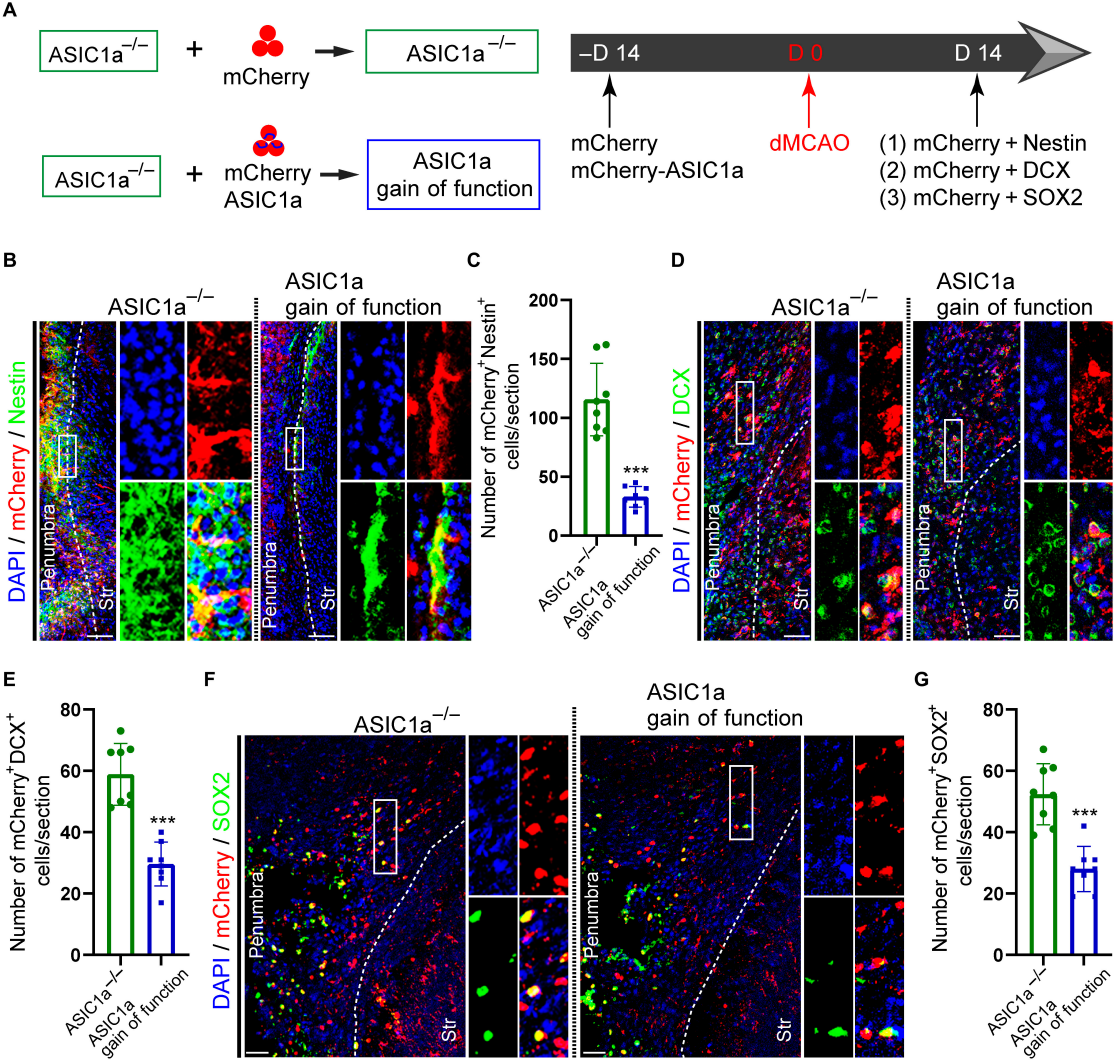
ASIC1a gain of function suppresses NPC migration toward penumbra and neurogenesis around infarct after ischemic stroke. (A) Experiment design and timeline. The ASIC1a*^−/−^* mice were received AAV-CMV-mCherry-2A-ASIC1a virus through LV injection to build ASIC1a gain-of-function mice on day 14 before dMCAO. TTC staining was performed on day 1 post-dMCAO. Immunostaining was conducted on day 14 to evaluate the migration of NPCs toward infarct. (B and C) Representative images of colocalization of Nestin and mCherry (B), and quantitation (C) illustrating the migration of NPCs from SVZ to penumbra on day 14. The insets presented the high magnification of Nestin^+^mCherry^+^ cells from the rectangle between the penumbra and striatum in each group. Scale bars, 20 μm. (D and E) Typical images of colocalization of DCX and mCherry (D), and quantification (E) demonstrating the migrated NPCs around infarct. The insets showed the high magnification of DCX^+^mCherry^+^ cells from the rectangle in penumbra per group. Scale bars, 20 μm. (F and G) Representative images of colabeling SOX2 and mCherry (F), and quantification (G) presenting the migrated NPCs from SVZ to infarct. The insets exhibited the high magnification of SOX2^+^mCherry^+^ cells from the rectangle in penumbra per group. Scale bars, 20 μm. Str: Striatum. DAPI was applied to counterstain the nuclei. ***P < 0.001.

To confirm the effect of ASIC1a deletion on facilitating NPC migration and neurogenesis after ischemic stroke, AAV-CMV-mCherry-2A-ASIC1a virus were injected into the LV of ASIC1a*^−/−^* mice (Fig. S9A and B) on day 14 before dMCAO (Fig. 6A). The number of mCherry^+^ cells in ASIC1a gain-of-function mice was predominantly decreased along the route to penumbra at the distance from 3,000 to 4,000 μm, while that was obviously increased less than 1,000 μm away from LV (Fig. S11A and B), suggesting that ASIC1a gain of function abrogated the effect of ASIC1a deletion on promoting NPC migration from SVZ to penumbra, to some extent. Moreover, the immunostaining of several NPC markers with the tracing marker of mCherry was conducted to verify the migration of NPCs from SVZ to penumbra in ASIC1a*^−/−^* and ASIC1a gain-of-function mice (Fig. 6A). The amount of mCherry^+^Nestin^+^ cells in ASIC1a gain-of-function mice was evidently declined compared with that in ASIC1a*^−/−^* mice on day 14 post-dMCAO (Fig. 6B and C). Subsequently, the number of mCherry^+^DCX^+^ cells in ASIC1a gain-of-function mice was significantly decreased in comparison with that in ASIC1a*^−/−^* mice on day 14 post-dMCAO (Fig. 6D and E). In addition, the number of mCherry^+^Sox2^+^ cells in ASIC1a gain-of-function mice were obviously reduced than that in ASIC1a*^−/−^* mice on day 14 post-dMCAO (Fig. 6F and G). Collectively, these results indicate that ASIC1a gain of function suppresses NPC migration from SVZ to penumbra and neurogenesis in penumbra, providing evidence for the role of ASIC1a deletion in NPC migration and neurogenesis in penumbra after ischemic stroke.

### Pharmacological inhibition of ASIC1a facilitates NPC migration and neurogenesis at pH 6.5 in vitro

**Fig. 7.**
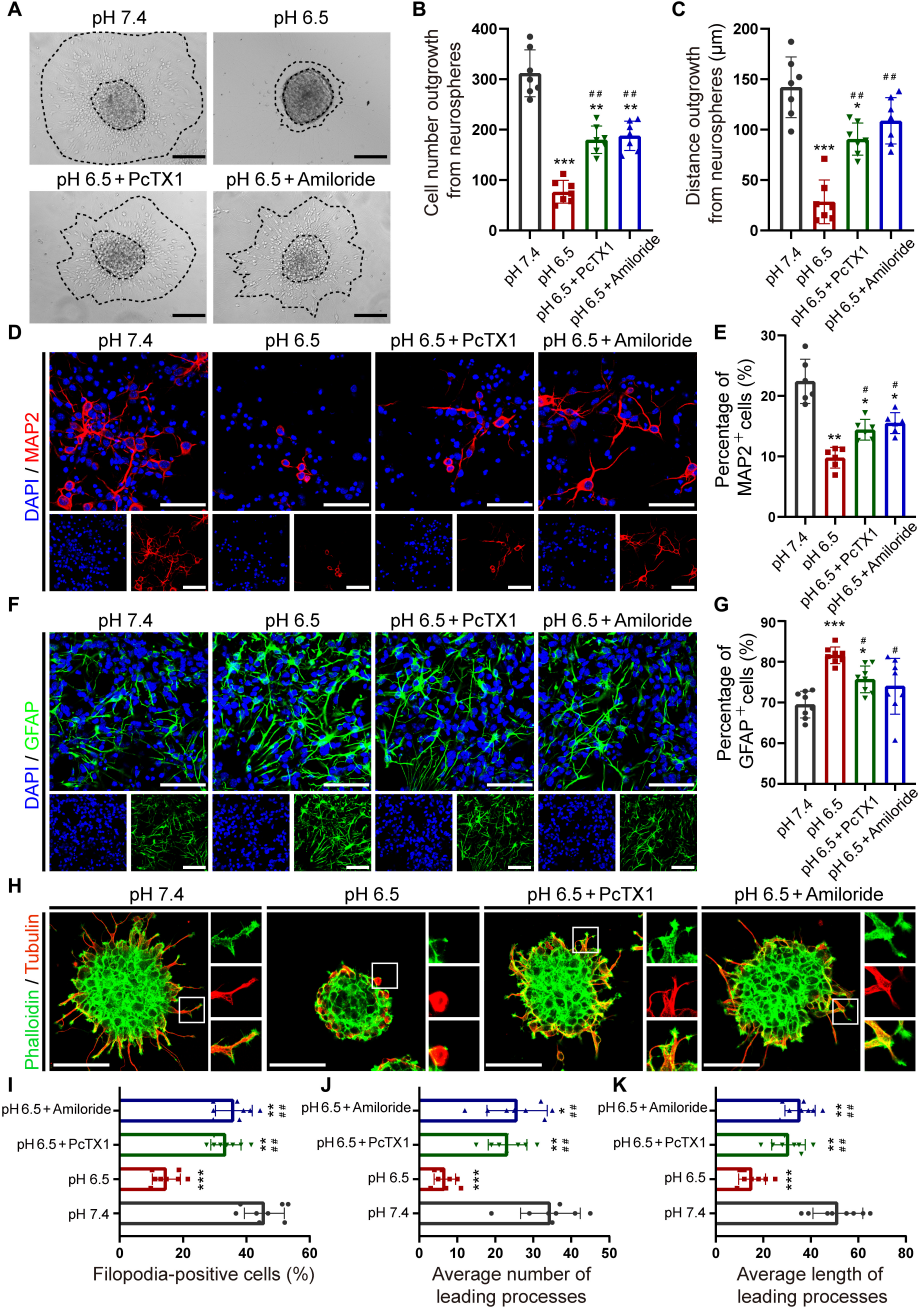
Pharmacological inhibition of ASIC1a facilitates NPC migration and differentiation into neurons under the acidic condition of pH 6.5 in vitro. (A to C) The migration of cultured neurospheres (A), and the migrating cell number (B) and distance (C) of NPCs under different conditions (pH 7.4, pH 6.5, pH 6.5 with amiloride/PcTX1) at 24 h. Scale bars, 100 μm. **P < 0.01, ***P < 0.001, compared with pH 7.4. ^##^*P* < 0.01, compared with pH 6.5. (D and E) Representative immunostaining images of MAP2 (D) and quantification of MAP2^+^ cells in each group (E). Scale bars, 10 μm. *P < 0.05, **P < 0.01, compared with pH 7.4. ^#^*P* < 0.05, compared with pH 6.5. (F and G) Typical immunostaining images of GFAP (F) and quantification of GFAP^+^ cells in each group (G). Scale bars, 10 μm. *P < 0.05, ***P < 0.001, compared with pH 7.4. ^#^*P* < 0.05, compared with pH 6.5. (H to K) Typical images of filopodia and leading processes formation (H), and quantitation of the average percentage of filopodia (I) and number (J) and length (K) of leading processes from (H) under different conditions (pH 7.4, pH 6.5, and pH 6.5 with amiloride/PcTX1). The square insets depicted the filopodia formation at the edge of cytoskeleton per group. DAPI was used to counterstain the nuclei. Scale bars, 20 μm. *P < 0.05, **P < 0.01, ***P < 0.001, compared with pH 7.4. ^##^*P* < 0.01, compared with pH 6.5.

The above results demonstrate that the inhibition of ASIC1a by genetic knockout of ASIC1a promotes NPC migration and differentiation into neurons. To investigate whether the inhibitors of ASICs, amiloride and PcTX1, could exert similar effects, we treated NPCs with amiloride (20 μM) or PcTX1 (100 ng/ml). The results demonstrated that the defective migration of NPCs at pH 6.5 was significantly ameliorated (Fig. 7A). Moreover, the number of migrating cells and distance out from neurospheres treated with amiloride or PcTX1 were obviously enhanced at pH 6.5 compared to pH 7.4 (Fig. 7B and C). Moreover, amiloride, as well as PcTX1, resulted in the preferential differentiation of NPCs into neurons (MAP2^+^) and decreased ratio of astrocytes (GFAP^+^) at pH 6.5 (Fig. 7D to G). Additionally, the percentage of filopodia formation was significantly increased by ASIC1a antagonist under the acidic condition of pH 6.5 (Fig. 7H and I). Simultaneously, the average number and length of leading processes treated with amiloride or PcTX1 were increased compared with control group in acidic environment (pH 6.5) (Fig. 7H, J, and K). Collectively, these results support the idea that pharmacological inhibition of ASIC1a facilitates NPC migration and differentiation into neurons under the acidic condition of pH 6.5 in vitro through facilitating filopodia formation.

### Pharmacological inhibition of ASIC1a improves NPC migration and behavioral recovery following ischemic stroke

**Fig. 8.**
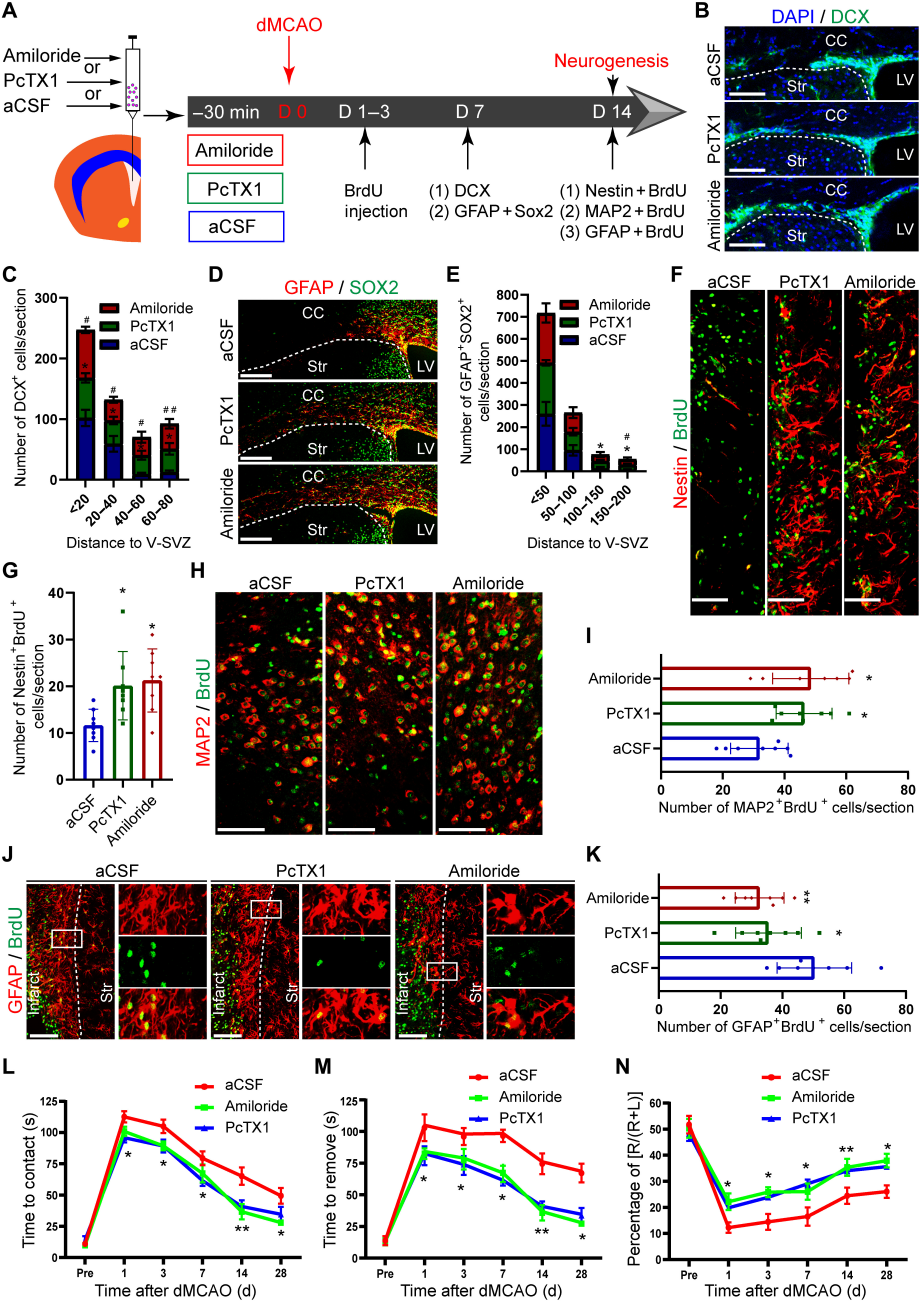
Pharmacological inhibition of ASIC1a facilitates NPC migration and neurogenesis following ischemic stroke. (A) Experiment design and timeline. The WT mice were received amiloride/ PcTX1/aCSF through LV injection 30 min before dMCAO. Immunostaining was performed to assess the migration of NPCs from SVZ to penumbra on day 7. Then, BrdU was administrated from day 1 to 3 post-dMCAO and immunostaining was implemented to evaluate the differentiation of migrated NPCs from V-SVZ to penumbra on day 14. (B and C) Representative immunostaining images of DCX (B) and quantification of DCX^+^ cells (C) in each group. Dotted lines indicated the margin of different brain regions. *P < 0.05, PcTX1 compared with aCSF. ^#^*P* < 0.05, ^##^*P* < 0.01, amiloride compared with aCSF. (D and E) Typical immunostaining images of GFAP and SOX2 (D) and quantification of GFAP^+^SOX2^+^ cells (E) in each group. Dotted lines represented the margin of different brain regions. *P < 0.05, PcTX1 compared with aCSF. ^#^*P* < 0.05, amiloride compared with aCSF. (F) Typical immunostaining images of Nestin^+^BrdU^+^ cells presenting the migrated NPCs from SVZ to infarct in each group. Scale bars, 20 μm. (G) Quantification of Nestin^+^BrdU^+^ cells in each group. (H and I) Representative immunostaining images of MAP2 and BrdU (H), and quantitation (I) showing the differentiation of migrated NPCs into newborn neurons around infarct. Scale bars, 20 μm. (J and K) Typical immunostaining images of GFAP and BrdU (J), and quantification (K) demonstrating the differentiation of migrated NPCs into newborn astrocytes around infarct. Dotted lines exhibited the margin of different brain regions. The insets showcase the high magnification of GFAP^+^BrdU^+^ cells from the rectangle in penumbra in each group. Scale bars, 20 μm. (L to N) Summarized curves demonstrating the adhesive removal test (L and M) and corner test (N) on days 1, 3, 7, 14, and 28. *P < 0.05, **P < 0.01. V-SVZ, ventricular-subventricular zone. DAPI was used to counterstain the nuclei.

Next, we assessed the effect of the above pharmacological inhibitors on NPC migration and neurogenesis in vivo. The WT mice were intraventricularly injected 2 μl of artificial cerebrospinal fluid (aCSF), aCSF-containing amiloride (300 μM) and PcTX1 (300 ng/ml) 30 min before dMCAO according to the methods previously reported by Xiong et al. [12] (Fig. 8A). Based on the previous study [26], the volume of CSF for adult mice is evaluated to be 40 μl. Assuming a uniform distribution of the infused drug in the CSF, we would expect that the concentration of amiloride was 15 μM and that of PcTX1 was 15 ng/ml, which was found to be effective in our cell culture experiments. We found that the number of DCX^+^ cells in mice treated with amiloride or PcTX1 was evidently increased along the route to penumbra at the distance from 40 to 80 μm, while that was obviously reduced less than 40 μm away from LV (Fig. 8B and C). Furthermore, the quantity of GFAP^+^SOX2^+^ cells in mice treated with amiloride or PcTX1 was profoundly increased along the path to penumbra at the distance from 100 to 200 μm (Fig. 8D and E). Then, the immunostaining of NPCs and BrdU among LV and along RMS was performed to test the effect of ASIC1a blockage on NPC migration under physiological conditions. The amount of BrdU^+^Nestin^+^ cells was evidently increased by intraventricular injection of ASIC1a blockers on day 14 post-dMCAO (Fig. 8F and G). Subsequently, the number of BrdU^+^DCX^+^ cells in the ASIC1a antagonist group was significantly reduced compared to that in the aCSF group on day 14 post-dMCAO (Fig. S11A and B). In addition, intraventricular injection of ASIC1a antagonist (amiloride or PcTX1) evidently increased the number of BrdU^+^SOX2^+^ NPCs on day 14 post-dMCAO (Fig. S11C and D). These results demonstrated that ASIC1a blockage promoted NPC migration from SVZ to penumbra after ischemic stroke in mice. Meanwhile, intraventricular injection of ASIC1a antagonist increased the number of BrdU^+^ MAP2^+^ cells around the injury region (Fig. 8H and I), whereas it decreased the number of BrdU^+^GFAP^+^ in penumbra (Fig. 8J and K). Finally, the adhesive removal test and the corner test were performed to explore whether the administration of amiloride or PcTX1 promoted behavioral recovery after dMCAO in mice (Fig. 8L to N). The results showed that mice treated with ASIC1a antagonist presented better behavioral recovery than WT mice. These results suggest that pharmacological blockage of ASIC1a promotes functional recovery by facilitating NPC migration and neurogenesis in penumbra after ischemic stroke in mice.

## Discussion

**Fig. 9:**
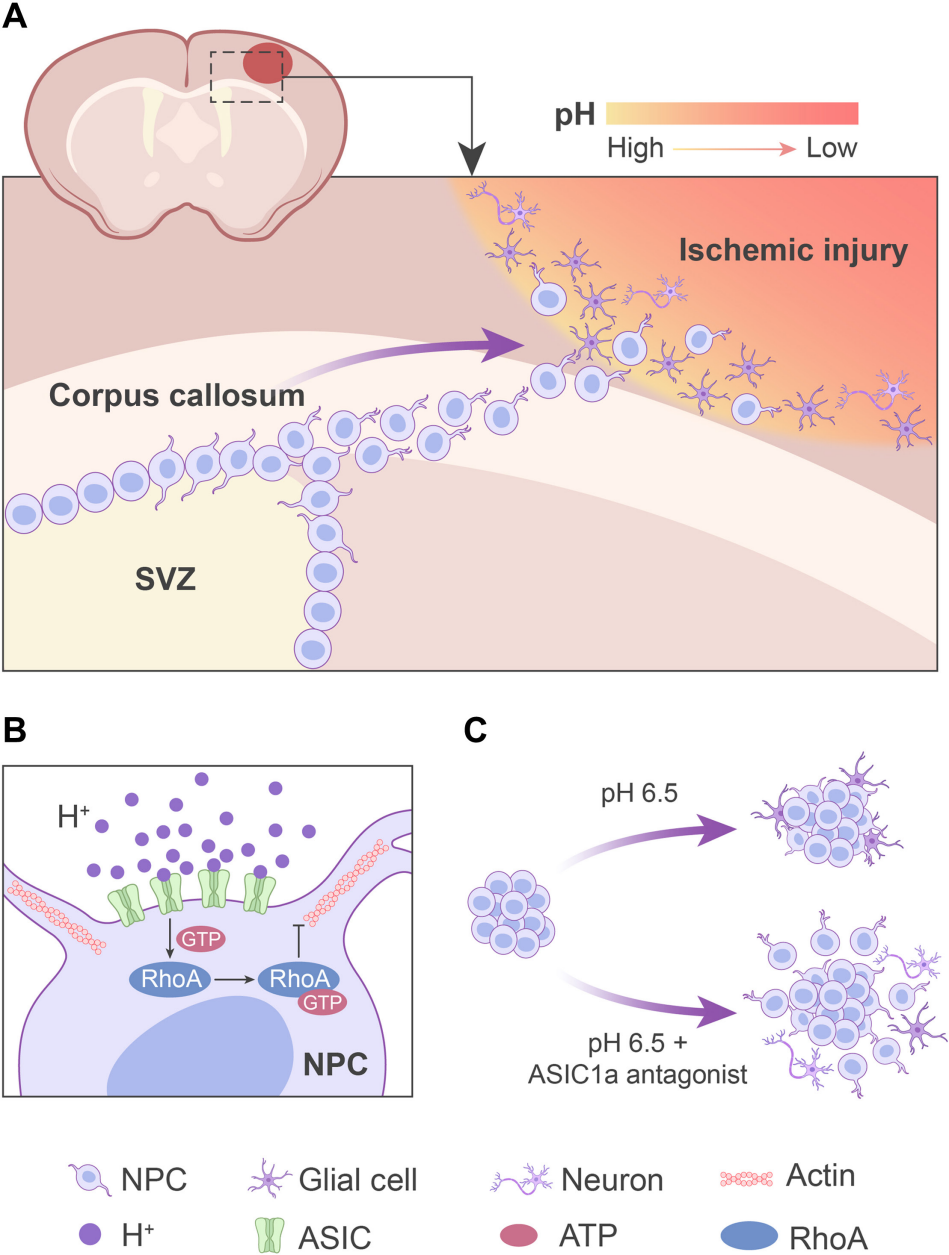
. Targeting ASIC1a facilitates NPC migration and neurogenesis in penumbra following ischemic stroke. (A) NPCs have the ability of migrating from the SVZ toward penumbra after ischemic stroke. (B) Activating ASIC1a by extracellular acidosis hinders NPC migration via triggering the RhoA signaling pathway to diminish cytoskeletal transformation and filopodia formation. (C) ASIC1 antagonists improves NPC migration by facilitating filopodia formation under the acidic condition of pH 6.5.

The present study demonstrates an essential role of ASIC1a in mediating migration and neurogenesis of NPCs. In this study, compelling genetic, physiological, histological, pathological, and molecular evidence shows that NPCs express ASIC1a with channel functionality. Activating ASIC1a by acidification impairs NPC migration and neurogenesis via triggering the RhoA signaling pathway to diminish cytoskeletal transformation and filopodia formation, which could be largely reversed by pharmacological or genetic disruption of ASIC1a. Notably, ASIC1a knockout promotes functional recovery by enhancing endogenous NPC migration toward the penumbra and infarct core after stroke, while ASIC1a gain of function partially abrogates this effect. Importantly, ASIC1a antagonists facilitate NPC migration and neurogenesis following ischemic stroke. Together, these findings suggest that targeting ASIC1a not only offers neuroprotection but also promotes neurorestoration after ischemic stroke, highlighting the importance of ASIC1a in the pathophysiology of CNS diseases (Fig. 9).

ASICs, as a main acid sensor in mammalian brain, can be specifically activated by microenvironmental acidification [14,16]. Of the ASIC subunits, ASIC1a is the surface protein in the brain with the most abundant expression [14], which is best known for its role in synaptic plasticity [27,28], mechanosensation [29], ischemic neuronal death [12,30,31], axonal degeneration [32], SCI [20], epilepsy, depression, and fear conditioning [33–35]. Here, our results show that ASIC1a is expressed in NPCs with channel functionality. Activating ASIC1a by acidification impairs NPC migration and neurogenesis in vitro and in vivo.

Thereafter, the bulk RNA-seq assay identifies RhoA as a pivotal downstream effector when activating ASIC1a under the acidic condition of pH 6.5. ASIC1a activation up-regulates active RhoA to suppress filopodia formation through modulating cytoskeletal organization, finally inhibiting NPC migration under the acidic condition of pH 6.5. Small guanosine triphosphatases play a crucial role in cytoskeleton arrangement and cell migration, and the constitutively active RhoA impedes cell motility [36,37], which is consistent with previous studies that the inhibition of RhoA signaling pathway enhances NPC migration and markedly attenuates the inhibitory effect of Slit2 on oligodendrocyte precursor cell migration [38,39]. Therefore, the present study discloses that RhoA is a novel downstream effector of ASIC1a.

Migration is a fundamental process of cell biology, but few studies pay attention to the role of ASICs in cell migration. Intriguingly, previous evidence suggests that ASICs are involved in the migration of glioma cells [40–42]. These studies reveal that ASIC silencing inhibits the migration of glioma cells. In contrast, our study reveals that ASIC1a activation represses NPC migration in vitro and in vivo. ASIC1a knockout prevents the defective migration of NPCs in acidic medium in vitro and enhances the migration of NPCs after stroke in vivo. However, reexpression of ASIC1a in cultured NPCs or in *ASIC1a^−/−^* mice reverses the effect of ASIC1a knockout on the migration of NPCs. Intriguingly, ASIC1a knockout does not affect the migration of NPCs in normal conditions both in vitro and in vivo. This finding is also backed up by the fact that no phenotype regarding neuronal migration and lamination deficiency during brain development has been reported in ASIC1 knockout mice [27,43]. This implies that blocking ASIC1a might affect the NPCs in the injured area associated with acidosis without effect on those in normal areas of the brain, providing an example of precise manipulation of NPCs in the target area.

Another important finding is that ASIC1a activation inhibits NPC differentiation into neurons. ASIC1a deletion facilitated neurogenesis on days 7 and 14, suggesting that ASIC1a deletion expedites neuroanatomical repair in the penumbra after ischemic stroke. This phenomenon might be explained by an increased number of migrated NPCs from SVZ to penumbra and their multiple effects such as cell replacement, immunomodulation, and the bystander effect of secreting various factors, including brain-derived neurotrophic factor, vascular endothelial growth factor, and epidermal growth factor [6,44]. Our results indicated that the number of migrating NPCs (DCX^+^ and GFAP^+^SOX2^+^ cells) from SVZ to penumbra was obviously increased after ASIC1a knockout or blockage. In particular, the number of GFAP^+^SOX2^+^ cells was evidently increased after ASIC1a knockout or blockage. Meanwhile, our results demonstrated that the number of GFAP^+^ cells was clearly increased without ASIC1a knockout or blockage in penumbra on day 14. The reason for this phenomenon might ascribe to in situ reactive astrogliosis, which are proliferated astrocytes activated by transforming growth factor-α, ciliary neurotrophic factor, interleukin-1, interleukin-6, and Kallikrein-related peptidase 6 in penumbra following ischemic stroke [45], while the number of neuroblast (DCX^+^ cells) and mature neurons was dramatically increased in penumbra after ischemic stroke, demonstrating that targeting ASIC1a was a feasible strategy to promote NPC migration and differentiation into neuron.

Electrophysiological characterization proves that functional ASICs are present in the NPCs. The ASIC-like current in cultured NPCs showed a high sensitivity to extracellular acidification. The currents are almost completely inhibited by amiloride, a commonly used inhibitor for the ASICs [11,46]. Moreover, PcTX1, which is capable of inhibiting homomeric ASIC1a and heteromeric ASIC1a/2b [47,48], represses the amplitude of the current by more than 50%. Therefore, the results show that the administration of amiloride and PcTX1 facilitates NPC migration and differentiation into neurons. It has been reported that pharmacological inhibition and genetic ablation of ASIC1a dramatically alleviate tissue damage after ischemic stroke [12,49,50]. As a diuretic and natriuretic that retains potassium, amiloride lowers the rates of K^+^, H^+^, Ca^2+^, and Mg^2+^ excretion. It is still unknown whether these effects play a role in the recovery of cerebral infarction [51]. At the same time, expression of ASIC1a is widely seen in the CNS and various types of immune cells [52]. It should be noted that most of ASIC1a studies have focused on the peripheral nervous system and CNS neurons. Although NPCs and immune cells play a critical role in damage progression after stroke, few studies focus on whether the resident population of ASIC1a or other ASICs in these cells leads to ischemic CNS injury. At the same time, a recent study demonstrates that amiloride improves myocardial fibrosis in cardioembolic stroke patient. To date, few studies have explored the effect of ASIC1a inhibition on patients with cerebral infarction [53]. Further studies are needed in this regard.

In summary, the present study demonstrates that ASIC1a activation elicits downstream of RhoA signaling to trigger reorganization of the cytoskeleton and filopodia formation, which ultimately impairs NPC migration and neurogenesis. ASIC1a deletion promotes functional recovery by enhancing NPC migration toward penumbra and neurogenesis after ischemic stroke. Pharmacological inhibition of ASIC1a has the same protective effect as the genetic knockout of ASIC1a. These results open up a novel research perspective regarding ASICs in NPCs and provide an innovative therapeutic strategy for increasing regenerative potential of NPCs during stroke and other neurological diseases with the presence of acidosis.

## Materials and Methods

### Mice

This research was approved by The Third Military Medical University Ethics Committee (approval no. SYXK 2012-0002) and followed the China’s animal welfare legislation for protecting animals used for scientific research. Every effort was made to minimize the number of animals and their sufferings. A total of 60 male mice (10 weeks old, 22 to 25 g; 48 mice were used for experiments, and 12 died during the experiments), 42 male *ASIC1a^−/−^* mice (10 weeks old, 22 to 25 g; 37 mice were used for experiments, and 5 died during experiments), and 9 heterozygous embryonic mice were used in the present study. All mice were housed on a constant photoperiod (12-h light/dark cycle) and temperature (22 to 25 °C) and at 55% to 60% moisture, and offered food and water ad libitum. All mice received anesthesia with 2% isoflurane/air mixture (1 to 2 l/min). A body temperature of 37 °C was kept using feedback-controlled heating system. All WT mice used in present experiments were littermates of *ASIC1a^−/−^* mice. *ASIC1a^−/−^* mice were purchased from the Jackson Laboratory (stock no. 013733).

### Primary NPC culture

Primary NPCs from the medial and lateral germinal eminences of C57BL/6 mice aged embryonic day 14.5 (E14.5) were obtained as previously described [23]. Briefly, the neocortices of pups from E14.5 C57BL/6 mice were gathered and washed using cold Hank's balanced salt solution (Beyotime, Beijing, China) at each dissection stage. Then, samples were incubated in 0.25% trypsin-EDTA (Thermo Fisher Scientific Inc., MA, USA) at 37 °C for 30 min. Afterward, the tissues were washed twice with Dulbecco’s modified Eagle’s medium (DMEM; Gibco, Grand Island, NY) together with soybean trypsin inhibitor (2.8 mg/ml, Thermo Fisher Scientific Inc., MA, USA). Thereafter, a fire-polished Pasteur pipette was used to triturate the samples, which were passed through a 100-μm Nylon cell strainer (BD Falcon, MA, USA) after being washed twice with DMEM. Cell suspensions were cultured in DMEM/F12 medium (Gibco, Grand Island, NY) in the presence of B27 (Gibco, Grand Island, NY), 20 ng/ml epidermal growth factor (PeproTech, Rocky Hill, NJ), and 20 ng/ml fibroblast growth factor-basic (PeproTech, Rocky Hill, NJ) at 37 °C in humidified atmosphere with 5% CO_2_. For cell passage, neurospheres were harvested after being centrifugated (100 × g), dissociated in StemPro Accutase Cell Dissociation Reagent (Gibco, Grand Island, NY), and cultured in medium as described above. For migration assays, the cell culture clusters were precoated with 10 μg/ml PLO based on previous protocols [23]. For pharmacological blockage, 20 μM amiloride (Sigma-Aldrich, St. Louis, MO, USA), 100 ng/ml PcTX1 (Peptide Institute, Inc., Osaka, Japan), or 5 μM Y27632 was added to the culture medium.

NPCs isolated from *ASIC1a^+/+^*, *ASIC1a^+/−^*, and *ASIC1a^−/−^* embryos, heterozygous embryonic mice were euthanized on day 14.5 after successfully mating with heterozygous adult male mice. Then, embryos were placed individually and each sample was placed in separate culture dish. After the culture procedures were finished as mentioned above, each tail from separate embryo was respectively collected for genotyping identification using standard polymerase chain reaction (PCR) method as described in the instructions of the manufacturer.

### RNA-seq sample preparation, data acquisition, and analysis

We generated a dataset of 3 biological replicates of paired primary NPCs, and samples were incubated in acidic condition for 24 h (pH7.4 and pH 6.5). Then, the samples were sent to a BGI Genomics Illumina platform (Shenzhen, China) for RNA-seq library construction and sequencing using an Illumina HiSeq 2000 sequencing platform.

We analyzed transcriptome profiling of pH 6.5 and pH 7.4 (control) in order to understand which genes might be critical to cell growth and migration in mice. The raw clean RNA-seq data were mapped to Mouse genome mm10 via SATR (v 2.7.1a) method in RSEM (v1.3.1) package, and the Bayesion adjusted count values were used for DEG analysis by DESeq2 (v1.28.1). As selected RAS pathway was important in pH decreasing, we selected 38 RAS pathway-related DEGs with Padj < 0.2 and abs| logFC | > 0.5, and 125 DEGs with Padj < 0.01 and abs| logFC | > 1.

### In vitro cytotoxicity assay

Based on manufacturer’s instructions, the release levels of LDH were determined by an LDH assay kit (Nanjing Jiancheng bioengineering Inc., Nanjing, China) to evaluate cytotoxicity. First, the respective supernatants were collected, and 2% Triton X-100 was used to lyse neurospheres for 15 min to release all LDH from the cytoplasm at 6, 12, and 24 h. A positive control was LDH released from cell lysates, which was thought to be the maximal LDH release. The amount of LDH release was determined by a microplate reader (Thermo Scientific, MA, USA) at the wavelength of 450 nm, and the results were shown as a ratio of LDH released in the medium to total cellular LDH.

### Cell viability assay

The cell viability was investigated by a CCK8 (Dojindo, Tokyo, Japan), which could quantify the live cell number by the use of a water-soluble tetrazolium salt and by production of an orange formazan dye upon bioreduction when an electron carrier was present. Briefly, 100 μl of cell suspension (1 × 10^5^ cells per well) was dispensed in a 96-well cell culture cluster under different conditions for 24 h and then incubated in 10% (v/v) CCK8 solution at 37 °C for 2.5 h. Then, the culture medium absorbance at a test wavelength of 450 nm was measured using a microplate reader (Thermo Scientific, MA, USA) as well as a reference wavelength of 630 nm.

### Flow cytometry assay

Apoptotic NPCs were detected by flow cytometry using an Annexin V-fluorescein isothiocyanate/PI apoptosis detection kit (Becton Dickinson, Franklin Lakes, NJ). Neurospheres incubated in different pH culture medium were collected, and dissociated using StemPro Accutase Cell Dissociation Reagent according to the recommendation of the manufacturer at 6, 12, and 24 h. After incubation with Annexin V-fluorescein isothiocyanate staining solution on ice for 10 min, cell resuspension and incubation were performed in binding buffer. Then, cell suspension was incubated in PI staining solution. After incubation for 5 min on ice in the dark, cell suspension was analyzed using a FACScan flow cytometer (Becton Dickinson, Franklin Lakes, NJ).

### Reverse transcription PCR

Total RNA extraction from neurospheres was performed using TaKaRa MiniBEST Universal RNA Extraction Kit (TaKaRa, Tokyo, Japan) based on the manufacturer’s instructions, and the contaminating DNA was removed using RNase-free DNase (Qiagen, Valencia, CA). Reverse transcription of 2 μg of RNA into complementary DNA (cDNA) was conducted using PrimeScript II 1st Strand cDNA Synthesis Kit (TaKaRa, Tokyo, Japan). An aliquot of cDNA mixture (0.2%) was chosen as templates for PCR. For PCR, the annealing temperature was 55 °C, and 30 cycles were performed. Gels were imaged using ChemiDoc XRS^+^ System (Bio-Rad California, USA). Primer sequences are listed in Table S1, Supplementary Materials.

### Western blot

Neurospheres were rinsed twice with ice-cold phosphate-buffered saline (PBS and homogenized with 200 μl of ice-cold RIPA (Sigma-Aldrich, St. Louis, MO) containing protease inhibitor cocktail (Roche, Indianapolia, IN, USA). Then, cell lysate was collected and centrifuged (10,000 × g) at 4 °C for 20 min. The concentration of protein was measured using an enhanced bicinchoninic acid protein assay kit (Beyotime, Beijing, China). Proteins (15 μg per lane) were separated using sodium dodecyl sulfate polyacrylamide gel electrophoresis on a 10% gel under reducing conditions and electroblotted to polyvinylidene difluoride membranes (Roche, Indianapolia, IN, USA). Afterward, the membranes were blocked in 5% (w/v) nonfat dry milk in tris-buffered saline (TBS) with Tween 20 (TBST) at room temperature for 2 h. Thereafter, the membranes were incubated in primary antibodies at 4 °C for 16 to 18 h. After being rinsed 3 times with TBST, the membranes were incubated in the corresponding horseradish peroxidase-conjugated secondary antibodies at room temperature for 2 h. Bands were washed with TBST and visualized with a chemiluminescence reagent kit (Beyotime Institute of Biotechnology) using a ChemiDoc XRS+ imaging system (Bio-Rad Laboratories, Inc.) and semiquantified using Image Lab software (version 2.0.1; Bio-Rad Laboratories, Inc.). Internal control was glyceraldehyde-3-phosphate dehydrogenase or β-actin for normalizing the expression level of each protein. The following antibodies were used: ASIC1 (27235-1-AP, Proteintech Group, Inc, Beijing, China), ASIC1 (ASC-014, Alomone, Jerusalem, Israel), ASIC2a (ASC-012, Alomone, Jerusalem, Israel), Tubulin (sc-73242, Santa Cruz Biotechnology, CA, USA), active RhoA (26904, Neweast, Bath, UK), RhoA (2117, Cell Signaling Technology, Danvers, MA), glyceraldehyde-3-phosphate dehydrogenase (sc-32233, Santa Cruz Biotechnology, CA, USA), and ASIC1a (MMS-5261, Biolend, San Diego, CA).

### Immunofluorescence

For immunofluorescence, 4% paraformaldehyde was used for fixing neurospheres or 25-μm brain frozen sections in 0.01 M PBS (~pH 7.4) at room temperature for 2 h, and then samples were blocked using normal goat serum mixed with 0.5% (v/v) Triton X-100 in PBS after being thoroughly washed. Then, the samples were incubated in primary antibodies at 4 °C for 16 to 18 h. On the next day, the specimens were incubated in corresponding Alexa Fluor 555- or Alexa Fluor 488-conjugated secondary antibody at room temperature for 2 h after being washed with PBS. Cell nuclei were counterstained with 4′-6-diamidino-2-phenylindole (DAPI) at room temperature for 15 min. Then, coverslips were placed on glass slides, and the images were obtained using a confocal microscope (LSM780, Carl Zeiss, Weimar, Germany) and examined using Zen 2011 software (LSM780, Carl Zeiss, Weimar, Germany). The following antibodies were adopted in this study and the ASIC1a antibody used in this study has been accepted and used in many published studies, and its specificity has also been recognized [9,20,54–57]: Nestin (ab-11306, Abcam, Cambridge, UK), Nestin (sc-21248, Santa Cruz Biotechnology, CA, USA), Tubulin (sc-73242, Santa Cruz Biotechnology, CA, USA), BrdU (MAB4072, Millipore, MA, USA), DCX (ab-18723, Abcam, Cambridge, UK), DCX (AB2253, Millipore, MA, USA), ASIC1 (sc-13905, Santa Cruz Biotechnology, CA, USA), ASIC1a (ASC-014, Alomone, Jerusalem, Israel), ASIC1a (MABN462, Millipore, MA, USA), Tubulin (sc-73242, Santa Cruz Biotechnology, CA, USA), DCX (4604, Cell Signaling Technology, Danvers, MA), DCX (ab-18723, Abcam), Nanog (8822, Cell Signaling Technology, Danvers, MA), and Sox2 (23064, Cell Signaling Technology, Danvers, MA) .

Brain sections were firstly incubated in 2 N HCl at 37 °C for 30 min for BrdU immunostaining, and then samples were incubated in 0.1 M borate solution (pH 8.5) twice for 10 min. Afterward, the specimens were submerged in 3% H_2_O_2_ for 30 min and blocked with 5% normal goat serum at room temperature for 1 h.

Cell counts were made using a minimum of 7 evenly spaced sections for each animal. To analyze migration, the peri-infarct and infarct core on confocal projection images were procured from 25-μm-thick coronal slices. Cells were counted in 4 slices per brain for calculating the relative cell percentages in the 2 areas. The quantification of MAP2^+^, GFAP^+^, Nestin^+^, DCX^+^, and BrdU^+^ cells was performed manually using the ImageJ multipoint tool and expressed as number of positive cells per cubic millimeter of tissue. Distance to ventricular-SVZ (V-SVZ) defined as the shortest distance between the edge of cell and the ependyma of the ventricles.

### Patch-clamp recording

For patch-clamp recordings, the cultured NPCs were used to record ASIC currents using whole-cell patch clamp. The pipette solution for whole-cell patch-clamp recordings contained 140 mM CsF, 10 mM Hepes, 11 mM EGTA, 2 mM tetraethyl ammonium chloride, 1 mM CaCl_2_, 2 mM MgCl_2_, and 4 mM K_2_ATP (~pH 7.3; 300 mOsm). The standard extracellular bath solution for whole-cell recording were listed as follows: 140 mM NaCl, 5.4 mM KCl, 2.0 mM CaCl_2_, 1.0 mM MgCl_2_, 20 mM Hepes, and 10 mM glucose (~pH 7.4; 320 to 330 mOsm). We prepared the acidic pH solutions based on previous description [12]. Briefly, 10 mM Hepes was used in the solutions at pH 7.4, pH 7.0, and pH 6.5, and 10 mM MES was used in the solution at pH ≤6.0. All chemicals were purchased from Sigma-Aldrich. PcTX1 was purchased from Peptides Institute. Mostly, we analyzed the current amplitude instead of current density. As for the analysis of current amplitude, cells with “run down” of greater than 20% of the original amplitude were not analyzed. For the administration of PcTX1, PcTX1 were preapplied for 20 s. In the experiment to verify that the transient inward currents could be blocked by ASIC1a inhibitors, PcTX1 were present in the acid solution, while in the reverse validation experiments, PcTX1 were washed out.

### Time-lapse phase-contrast observation

Time-lapse phase-contrast images of neuroblasts migrating out of neurospheres were obtained using an EVOS FL Auto Cell Imaging System (Thermo Scientific, MA, USA) every 10 min for up to 24 h using a 20× objective in a controlled atmosphere (37 °C, 5% CO_2_). The isolated neurospheres with a diameter >35 μm and cells remaining in focus during the 24 h were used for analyses.

### NPC migration assay

For neurosphere migration assay, neurospheres were seeded on PLO-precoated 24-well cell culture clusters, and images were captured using a phase-contrast microscopy at 10× magnification once every 1 h for 10 h allowing for the tracking of NPC migration out of the neurospheres. The phase-contrast images (*n* = 6 per sample well) were analyzed using a custom-designed MATLAB program (MathWorks, Inc., Natick, MA) and were normalized to baseline measurements 2 h after being plated [23]. All neurosphere experiments in 4 wells were conducted, and results of at least 3 independent experiments were shown.

For transwell assay, insert membranes were coated with growth factor-reduced reconstituted extracellular matrix gel (Corning, Tewksbury, MA) containing enrichment medium at a 1:7 ratio. A total of 100 μl (1 × 10^5^) NPCs were seeded in the upper chambers, while 600 μl of DMEM/F-12 medium supplemented with 10% fetal bovine serum filled the lower chambers as a chemoattractant. The NPCs were allowed to move from the upper to lower chambers at 37 °C for 24 h in a humidified incubator with 5% CO_2_. Nonmigratory cells were softly moved away from the top of the membrane using a cotton swab. After being fixed for 30 min in 4% paraformaldehyde at room temperature, cell staining on the lower surface of membrane was done with 0.1% crystal violet at 37 °C for 30 min, and then the cells were counted under a microscope using a 20× objective. For each transwell filter, 5 fields were counted.

### SVZ explants migration assay

SVZ explants were procured from E14.5 C57BL/6 mice as previously described [58] and mixed with Matrigel containing enrichment medium at a 3:1 ratio. The explant–Matrigel mix was seeded on the surface of cell culture cluster and incubated at 37 °C in a humidified incubator with 5% CO_2_ (or until the solidifying of Matrigel). Then, medium at different pH was added to observe migration chain at 37 °C in a humidified incubator with 5% CO_2_. The medium was changed every 6 h to maintain the stability of pH fluctuation. For explant migrating area calculation, cells that migrated out from explants formed bright regions surrounding the explants in phase-contrast images (4× magnification, Olympus IX71). The migration area was quantified by measuring the area of a bright region around an explant, which were shown after normalization to the explant perimeter (Axiovision software v4.1, Zeiss, Thornwood, NY) [58].

### Filopodia detection

For visualization of filopodia formation, neurospheres were incubated in Alexa Fluor 488-conjugated phalloidin reagents at room temperature for 30 min. Then, samples were covered with the ProLong Gold Antifade Reagent with DAPI, and the images were taken by a confocal microscope (LSM780, Carl Zeiss, Weimar, Germany) and investigated using Zen 2011 software (LSM780, Carl Zeiss, Weimar, Germany).

### Transfection

ASIC1a-specific siRNA, RhoA-specific siRNA, and ASIC1a-specific CRISPR were obtained from Santa Cruz Biotechnology. Transfection of siRNA or ASIC1a-specific CRISPR was conducted using Lipofectamine RNAiMAX transfection reagent according to the manufacturer’s instructions. The same amount of Lipofectamine RNAiMAX was used as negative control. Afterward, the expression of target proteins was assessed by Western blot assays.

### dMCAO

Mice were anesthetized with 2% isoflurane/air mixture (1 to 2 l/min). Body temperature was kept at 37 ± 0.3 °C by a feedback-controlled heating system (Zhongshi, Inc., Beijing, China) during surgery. The surgical procedures for dMCAO were performed according to previous reports [59]. Briefly, under the surgical microscope, a horizontal incision of 4 mm was made in the skin between the left orbit and the auditory canal. Afterward, a hole of 2-mm diameter directly over the MCA was made using a micro drill. Forceps were used to remove the meninges and then using a small vessel cauterizer, MCA is permanently cauterized at a point downstream of the lenticulostriate branches.

### LV injection of ASIC1a antagonists and adeno-associated virus for ASIC1a reexpression

For LV injection of ASIC1a antagonists, 2 μl of aCSF (vehicle) alone, aCSF-containing amiloride (300 μM), or PcTX1 venom (300 ng/ml) was intraventricularly injected into the LVs (0.5 μl/min) using specific coordinates (0.2 mm posterior to bregma, 2 mm ventral to the skull, and 1 mm lateral to the sagittal line) with a stereotaxic frame (Zhongshi, Inc., Beijing, China) 30 min before and after the ischemia after mice (*n* = 4 for each group) were anesthetized.

For ASIC1a reexpression, intraventricular administration of 2-μl AAV-CMV-mCherry-2A or AAV-CMV-mCherry-2A-ASIC1a into the LVs (0.33 μl/min) was performed using specific coordinates (0.2 mm posterior to bregma, 2 mm ventral to the skull, and 1 mm lateral to the sagittal line) with a stereotaxic frame (Zhongshi, Inc., Beijing, China) 14 d before dMCAO surgery after mice (*n* = 5 for each group) were anesthetized. The efficiency of ASIC1a overexpression in LV-SVZ was determined using Western blot assays and immunostaining.

### BrdU injections

To study the proliferation of NPCs, 1 intraperitoneal injection of 50 mg/kg BrdU was given to a cohort of mice 2 h before perfusion and sacrifice (*n* = 5 for both *ASIC1a^+/+^* and *ASIC1a^−/−^*). To investigate how NPCs migrated, 3 intraperitoneal BrdU injections (50 mg/kg) were given to another cohort of mice per day for 3 d consecutively, and the mice were sacrificed 4 d after the last injection (*n* = 5 for both *ASIC1a^+/+^* and *ASIC1a^−/−^*).

### Triphenyltetrazolium hydrochloride (TTC) staining

TTC staining was performed as previously described [12]. Briefly, all mice were euthanized after being anesthetized with 2% isoflurane/air mixture (1 to 2 l/min). Brains were immediately taken away, coronally sectioned at 1- to 2-mm intervals, and stained with 2% TTC. Calculation of infarction area involved subtracting the normal area stained with TTC in the ischemic hemisphere from the area of the nonischemic hemisphere. The calculation of infarct volume was performed by summing infarction areas of all sections and multiplying by slice thickness.

### Adhesive removal test

Each animal paw was applied with 2 strips of adhesive tape (0.3 * 0.4 cm^2^) with equal pressure. Between each animal and each session, the adhesive placement order (right or left) was alternated. After placing the mouse in a transparent box, we collected the times to contact and to remove each strip of adhesive tape with a maximum of 120 s. One trial per testing day was conducted. Prior to the surgery, mice received training every day, and after surgery, they received regular test. Performances of the last 3 d of each week served as a block on average [24].

### Corner test

In the corner test, a mouse was placed between 2 boards (30 × 20 × 1 cm each). The edges of the 2 boards were attached at a 30° angle with a small opening along the joint placed between the 2 boards to encourage the mice to enter the corner. At the beginning of a trial, the mouse was placed between the 2 angled boards facing the corner. When the mouse entered into the corner, the vibrissae on both sides were stimulated simultaneously. After MCAO, animals with unilateral brain damage would exhibit unidirectional turning. Each mouse was tested for 20 trials, and the side to which it turned (right or left) was recorded. The data were expressed as the percentage of right turns / total number of turns, and then these were statistically analyzed.

### Data analysis

Data were presented as means ± SEM, and SPSS v19.0 (IBM Corp.) was used for statistical analyses. One-way ANOVA followed by Tukey’s post hoc test was adopted for statistical comparison between groups. A Student *t* test was employed for 2-group comparison. Dose-response curve was fitted using OriginPro 8 software with an equation of *E*  =  Emax {1/[1 + (EC_50_/*C*)*n*]}, in which *E* represents effect at concentration *C*, Emax the maximal effect, EC_50_ the concentration for half-maximal effect, and *n* the Hill coefficient. The analyses did not constrain the Hill coefficient value. A statistically significant difference was considered to be at *P* < 0.05.

## Data Availability

The datasets presented in this article are not readily available because the project is still ongoing, but data will be available at a later date on reasonable request to the principal investigators.
