# Supplementary material for: Targeting ASIC1a Promotes Neural Progenitor Cell Migration and Neurogenesis in Ischemic Stroke
Source: Research (Wash D C). 2023 Jun 1;6:0105. doi: 10.34133/research.0105 (PMC10234266; doi:10.34133/research.0105)
Supplement: Supplementary 1 — Figs. S1 to S11 [file research.0105.f1.pdf]

Supplementary Information

Targeting ASIC1a Promotes Neural Progenitor Cells Migration and Neurogenesis in Ischemic Stroke

Hongfei Ge, Tengyuan Zhou, Chao Zhang, Yupeng Cun, Weixiang Chen, Yang Yang, Qian Zhang, Huanhuan Li, Jun Zhong, Xuyang Zhang, Hua Feng, Rong Hu,

Supplementary Figure 1

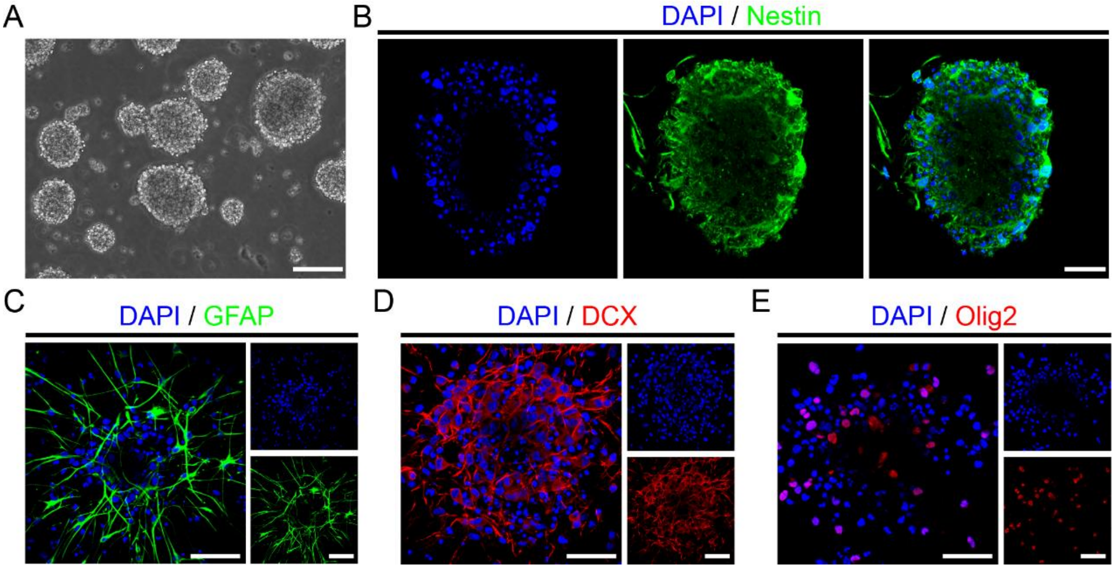

Figure S1. Primary NPCs culture and characteristics.

(A) The suspended neurospheres were grown in floating condition in enrichment medium on day 3. Scale bar, 100 μm. **(B)** Immunostaining images presenting most of cultured cells expressed Nestin. **(C-E)** Immunostaining images illustrating cultured cells held the multipotency of differentiation into $GFAP^+$ cells (C), DCX^+ cells (D) and $Olig2^+$ cells (E). Cell nuclei were counterstained with DAPI in blue. Scale bars, 20 μm.

Supplementary Figure 2

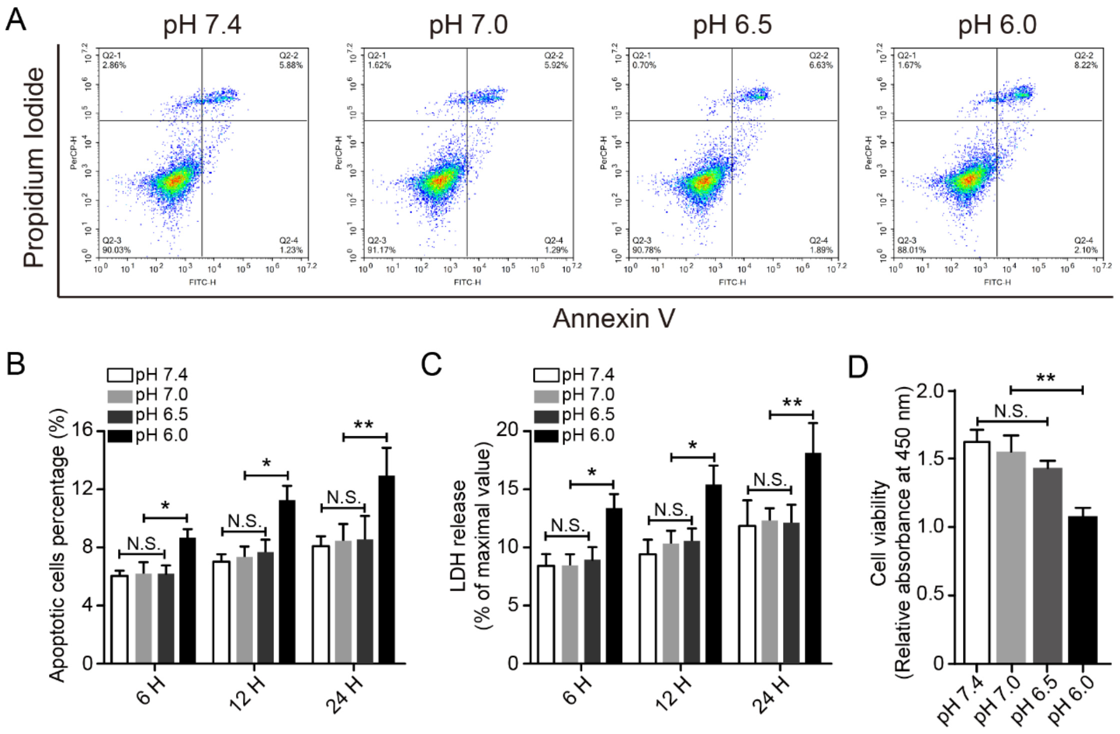

Figure S2. The effect of extracellular acidosis on survival and viability of NPCs. **(A)** Typical images showing death of NPCs under different pH condition through flow cytometry. **(B)** Quantitation from (A). **(C)** LDH assays under different pH condition at different time points. **(D)** Bar graph indicating cell viability under different pH conditions at 24 h. *p<0.05, **p<0.01; N.S., not significant.

Supplementary Figure 3

A

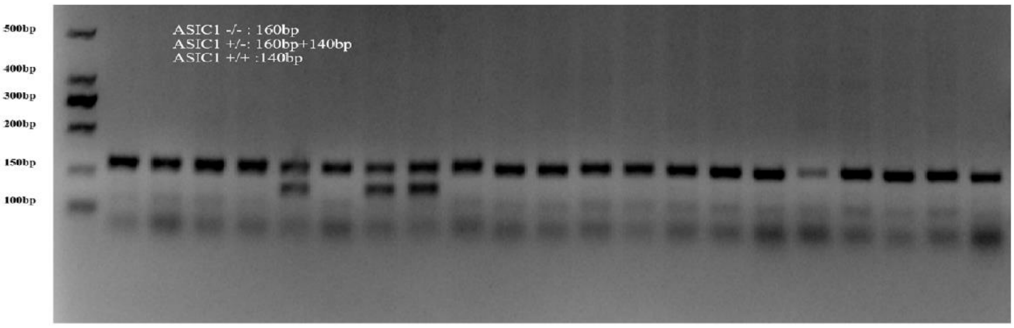

Figure S3. ASIC1a$^{-/-}$ mice were identified by PCR.

Supplementary Figure 4

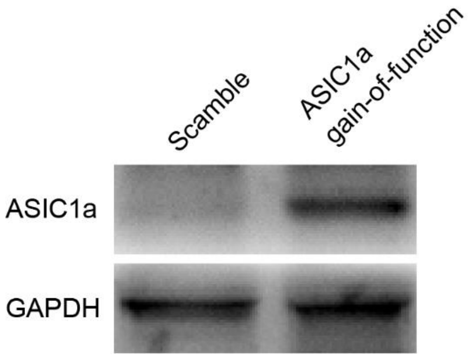

Figure S4. The expression of ASIC1a using ASIC1a-specific CRISPR to build ASIC1a gain-of-function NPCs.

Immunoblot bands depicting the expression of ASIC1a with ASIC1a-specific CRISPR transfection in vitro.

Supplementary Figure 5

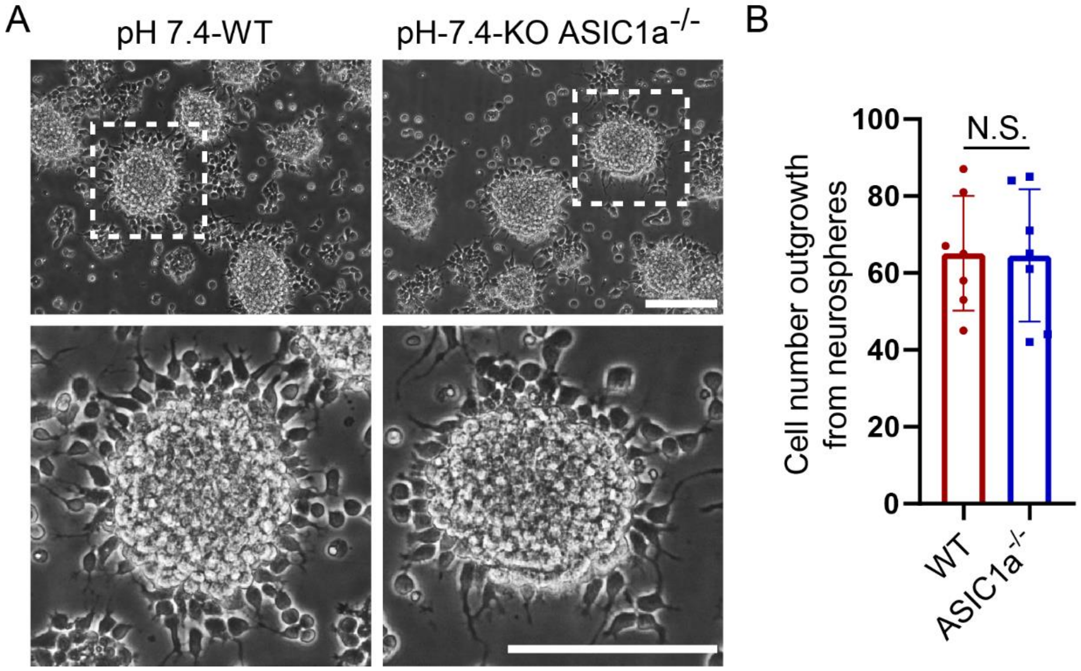

Figure S5. Neurospheres isolated from ASIC1a$^{-/-}$ mice exhibit no significant difference on migration under physiological condition of pH 7.4.

(A) The migration of cultured NPCs from ASIC1$^{+/+}$ and ASIC1$^{-/-}$ mice under pH 7.4. Scale bars, 100 µm. **(B)** Quantification of cell number migration from neurospheres from (A). N.S., not significant.

Supplementary Figure 6

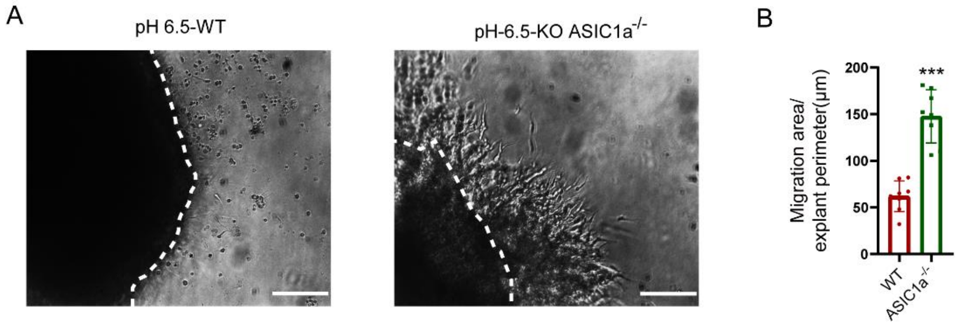

Figure S6. ASIC1a deletion enhances NPCs migration under pH 6.5 condition.

(A) Representative images of cell emigration from the SVZ explants (ASIC1$^{+/+}$ and ASIC1$^{-/-}$ mice) under pH 6.5 condition at 24 h. Scale bars, 100 µm. **(B)** quantitative analysis of cell emigration from the SVZ explants (ASIC1$^{+/+}$ and ASIC1$^{-/-}$ mice) under pH 6.5 condition at 24 h. $^{***}p<0.001$.

Supplementary Figure 7

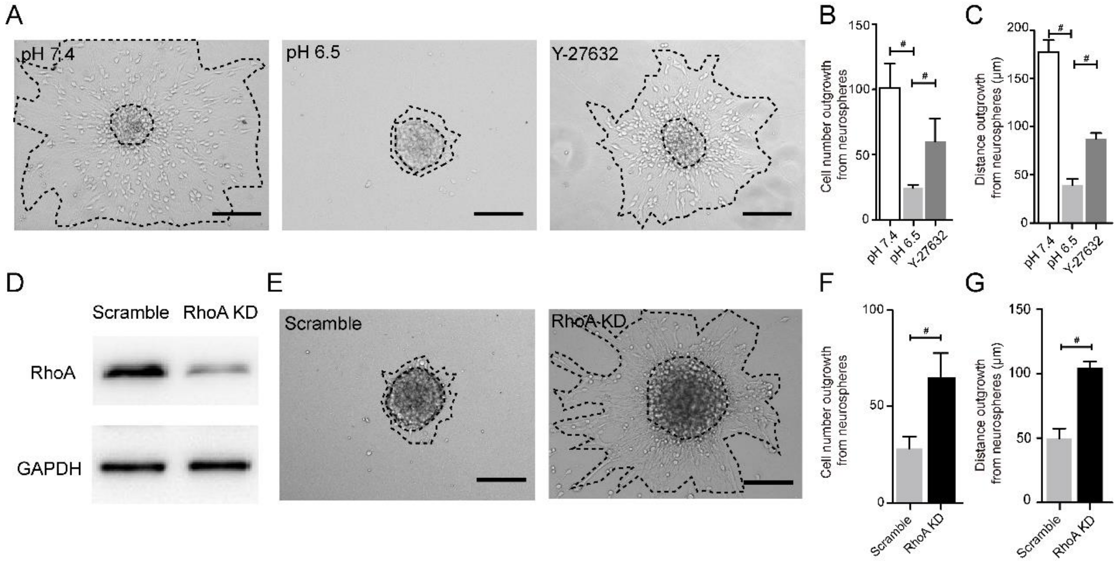

Figure S7. Inhibition of RhoA signaling pathway enhances NPCs migration under pH 6.5 condition

(A) Phase-contrast images showing the effect of RhoA inhibitor Y27632 on NPCs migration in different groups. Scale bar: 100 μm. **(B, C)** Bargraph summarizing the cell number and distance emigration from neurospheres from (A). $^{\#}p<0.01$. One-way ANOVA followed by Tukey's post hoc test. **(D)** The efficiency of RhoA Knockdown was verified by immunoblotting. **(E)** Phase-contrast images showing the effect of RhoA knockdown on the NPCs migration under acidic stimulation. Scale bar: 100 μm. **(F, G)** Bargraph summarizing the cell number and distance emigration from neurospheres from (E). $^{\#}p<0.01$.

Supplementary Figure 8

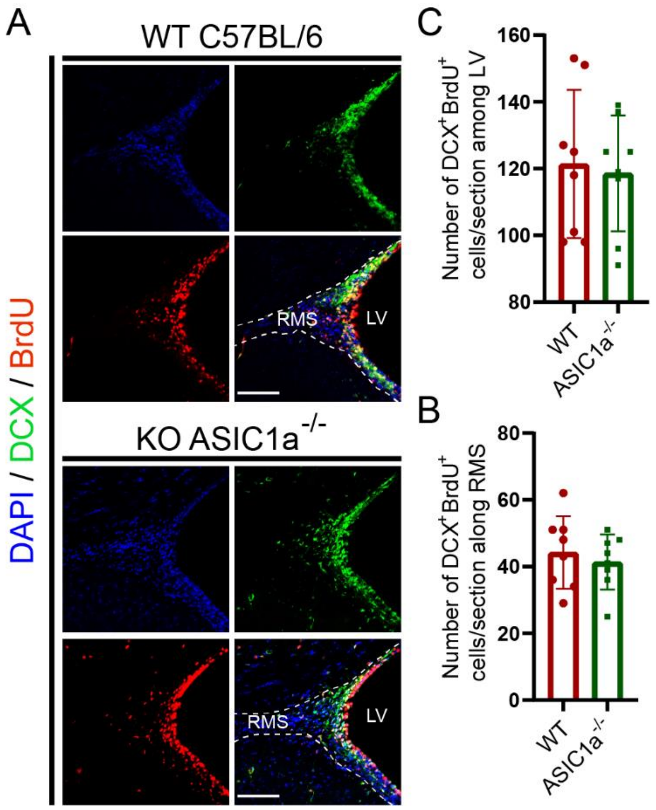

Figure S8. ASIC1a$^{-/-}$ mice exhibit no significant difference on proliferation of NPCs among LV and along RMS.

(A) Typical immunostaining images of DCX and BrdU presenting the number of NPCs among LV and along RMS. Scale bars, 20 μm. **(B** and **C)** Bar charts illustrating the average number of DCX$^+$BrdU$^+$ cells among LV (B) and along RMS (C) from (A) in each group.

Supplementary Figure 9

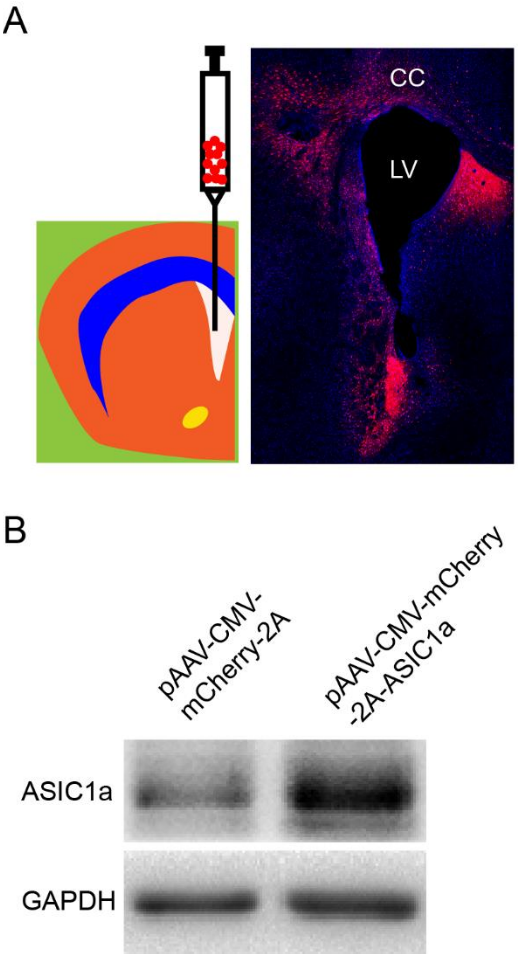

Figure S9. The expression of ASIC1a using pAAV-CMV-mCherry-2A-ASIC1a to build ASIC1a gain-of-function mice.

(A) The schematic illustration of pAAV-CMV-mCherry-2A-ASIC1a virus through LV injection to build ASIC1a$^{-/-}$ gain-of-function mice on day 14 before dMCAO. **(B)** Bands showing the expression of ASIC1a with pAAV-CMV-mCherry-2A-ASIC1a injection through LV.

Supplementary Figure 10

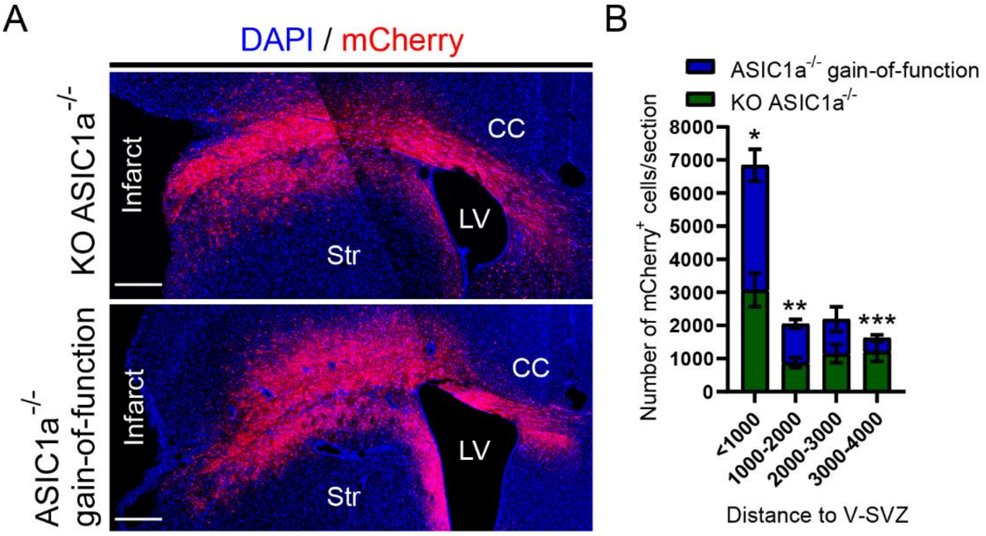

Figure S10. The expression of ASIC1a using pAAV-CMV-mCherry-2A-ASIC1a to build ASIC1a$^{-/-}$ gain-of-function mice.

(A) Representative images showing the migration of mCherry^{+} cells from SVZ to infarct. Scale bars, 100 μm. **(B) Q**uantitation of the number of mCherry^{+} cells from SVZ along RMS to infarct (A). $^{*}p<0.05$, $^{**}p<0.01$, $^{***}p<0.001$.

Supplementary Figure 11

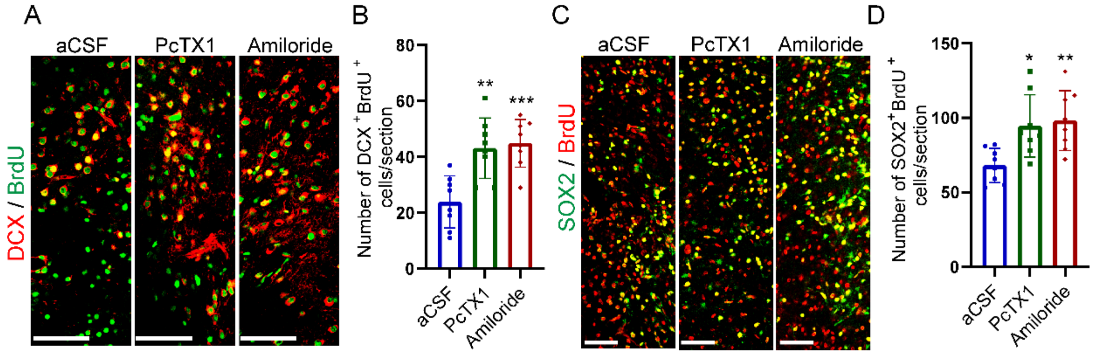

Figure S11 The ASIC1a antagonist facilitates NPCs migration

(A) Representative images showing the migration of $BrdU^{+}DCX^{+}$ cells from SVZ to infarct. Scale bars, 100 μm. **(B)** Quantitation of the number of $BrdU^{+}DCX^{+}$ cells from SVZ along RMS to infarct (A). **p<0.01, ***p<0.001. **(C)** Representative images showing the migration of $BrdU^{+}SOX2^{+}$ cells from SVZ to infarct. Scale bars, 100 μm. **(D)** Quantitation of the number of $BrdU^{+}SOX2^{+}$ cells from SVZ to infarct. *p<0.05, **p<0.01.
